# T-cell phenotype including CD57^+^ T follicular helper cells in the tumor microenvironment correlate with a poor outcome in follicular lymphoma

**DOI:** 10.1038/s41408-023-00899-3

**Published:** 2023-08-18

**Authors:** Zhi-Zhang Yang, Hyo Jin Kim, Hongyan Wu, Xinyi Tang, Yue Yu, Jordan Krull, Daniel P. Larson, Raymond M. Moore, Matthew J. Maurer, Kevin D. Pavelko, Shahrzad Jalali, Joshua C. Pritchett, Rekha Mudappathi, Junwen Wang, Jose C. Villasboas, Patrizia Mondello, Anne J. Novak, Stephen M. Ansell

**Affiliations:** 1 Division of Hematology and Internal Medicine, Mayo Clinic, Rochester, MN USA; 2 Department of Immunology, Medical College, China Three Gorges University, Yichang, Hubei China; 3 Department of Quantitative Health Sciences, Mayo Clinic, Rochester, MN USA; 4 Department of Pathology, Mayo Clinic, Rochester, MN USA; 5 Department of Immunology, Mayo Clinic, Rochester, MN USA; 6 Department of Quantitative Health Sciences and center for Individual Medicine, Mayo Clinic, Scottsdale, AZ USA; 7 College of Health Solutions, Arizona State University, Scottsdale, AZ USA

**Keywords:** Tumour immunology, B-cell lymphoma

## Abstract

T-lymphocytes are prevalent in the tumor microenvironment of follicular lymphoma (FL). However, the phenotype of T-cells may vary, and the prevalence of certain T-cell subsets may influence tumor biology and patient survival. We therefore analyzed a cohort of 82 FL patients using CyTOF to determine whether specific T-cell phenotypes were associated with distinct tumor microenvironments and patient outcome. We identified four immune subgroups with differing T-cell phenotypes and the prevalence of certain T-cell subsets was associated with patient survival. Patients with increased T cells with early differentiation stage tended to have a significantly better survival than patients with increased T-cells of late differentiation stage. Specifically, CD57^+^ T_FH_ cells, with a late-stage differentiation phenotype, were significantly more abundant in FL patients who had early disease progression and therefore correlated with an inferior survival. Single cell analysis (CITE-seq) revealed that CD57^+^ T_FH_ cells exhibited a substantially different transcriptome from CD57^−^ T_FH_ cells with upregulation of inflammatory pathways, evidence of immune exhaustion and susceptibility to apoptosis. Taken together, our results show that different tumor microenvironments among FL patients are associated with variable T-cell phenotypes and an increased prevalence of CD57^+^ T_FH_ cells is associated with poor patient survival.

## Introduction

The clinical course of follicular lymphoma (FL) varies, with some patients surviving much longer than others [1]. In addition, the response to treatment differs among patients with some achieving complete remission and others having disease progression [2] with a subset of patients who progress within the first 2 years having a poor outcome [3]. The underlying mechanism by which the disease course, response to treatment and survival of FL patients differs from each other is largely unknown.

While genetic, epigenetic, and biologic changes in malignant B cells are important factors in FL disease progression, the tumor microenvironment (TME) also plays a critical role by regulating antitumor immunity and thus impacting patient outcome [4–6]. The TME in FL consists of a variety of cell types including T cells, monocytes/macrophages and natural killer (NK) cells. These cells interact with malignant B cells and play an important role in the regulation of phenotype, differentiation, and growth of B cells. Among cell types in FL TME, T-cells are a predominant population constituting ~20% of mononuclear cells in the tumor. These T cells typically reside close to malignant B cells and interact each other with the malignant clone.

Our previous studies have identified multiple T-cell subsets in FL TME that have clinical significance [7–12]. These T subsets showed distinct phenotypes and we have shown that malignant B cells play an important role in the establishment of a tumor-friendly phenotype in T cells [13, 14]. As a result, these T cells partially or completely lose their anti-tumor function and promote a pro-malignancy TME [15]. In the present study, we evaluated a cohort of 82 FL patients and employed CyTOF to determine the phenotype of mononuclear cells. We grouped patients based on the numbers of major lineages and measured T-cell phenotypes in each patient group. We explored the differential effect of T cell phenotype on the survival in patients with follicular lymphoma grade1/2 compared to 3a/b. We further determined the role of CD57^+^ T_FH_ cells in treatment response and assessed transcriptome of this subset in FL using CITE-seq analysis.

## Results

### T-cell phenotype varies in patients with distinct tumor microenvironments in FL

**Fig. 1:**
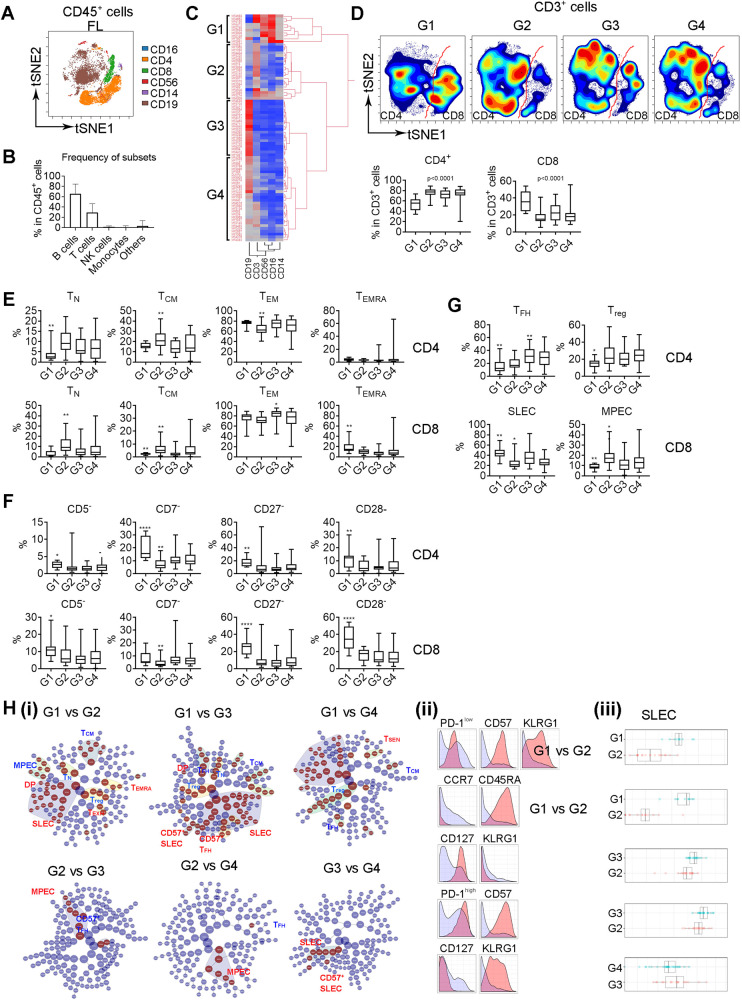
T-cell phenotype varies in patients with distinct tumor microenvironments in FL. **A** The tSNE plot showing expression and distribution of CD16^+^, CD4^+^, CD8^+^, CD56^+^, CD14^+^, and CD19^+^ in CD45^+^ cells from a representative FL biopsy specimen, *n* = 82. **B** Graph showing percentage of B, T, NK, monocytes, and others in CD45^+^ cells in FL biopsy specimens (*n* = 82). **C** Heatmap showing four patient groups clustered by the content of CD19^+^, CD3^+^, CD14^+^, CD56^+^, and CD16^+^ cells using hierarchical clustering analysis. **D** The tSNE plots of CD3^+^ subsets from four patient groups and the t-SNE analysis was performed on a concatenated file of each patient group. The red line was drawn to separate CD4^+^ and CD8^+^ T cells. Graphs below showing percentage of CD4^+^ and CD8^+^ cells in each group. The *p* value indicates a difference between G1 and G2, G1 and G3 or G1 and G4 in both CD4^+^ and CD8^+^ cells. **E**–**G** Graphs showing percentage of T_N_, T_CM_, T_EM_, and T_EMRA_ (**E**) as well as CD5^−^, CD7^−^, CD27^−^, CD28^−^ (**F**) in both CD4^+^ and CD8^+^ cells in each group. graphs in Figure G showing percentage of T_reg_ and T_FH_ in CD4^+^ and SLEC and MPEC in CD8^+^ cells in each group. The *p* value indicates a difference between group with * and the other groups. *<0.05; **<0.01; ***<0.001; ****<0.0001. **H** CITRUS plot showing clustering results from FL patients divided by twq patient groups. Circles in red represent clusters that differed between groups. Each class of clusters was annotated based on the phenotype. Histogram plots showing expression of selected markers by cells from clusters overlaid on background staining (i). Expression level of each selected marker was expressed by cluster (red) over background (light blue) (ii). Graph showing quantitative results of abundance from selected clusters (iii).

The tumor microenvironment (TME) in FL showed the cellular heterogeneity as we were able to identify 33 clusters (cell subsets) that constituted cell subsets from mononuclear cells including B cells, T cells, monocytes/macrophages and NK cells within a FL tissue sample (Supplementary Fig. 1A). To explore whether FL patients present variable intratumoral T-cell phenotypes, we evaluated a cohort of 82 FL patient tumor tissues that were collected before initial treatment. The patient characteristics were shown in Supplementary Table 1. Notably, some parameters have missing annotations resulting in decreased patient numbers. Mass cytometry (CyTOF) was performed with a staining panel focusing on T-cell-related markers (Supplementary Table 2). In this cohort, the frequency of monocytes/macrophages (CD14^+^) and NK cell (CD56^+^ or CD16^+^) was low (Fig. 1A, B). T cells were the dominant population, aside from lymphoma B cells (Fig. 1B). Expression level of all surface markers was summarized in Supplementary Fig. 1B.

Next, we tested whether the T-cell phenotype varies in patients with distinct TME in FL. Using hierarchical clustering, we stratified patients into four groups (Fig. 1C, G1–G4). Patients from G1 and G2 showed an increased number of monocytes/macrophages/NK cells and T cells, respectively. CD19^+^ B cells were enriched in G3 and patients with intermediate numbers of B and T cells were grouped into G4 (Supplementary Fig. 1C). Supporting this analysis, unsupervised clustering of the entire dataset in a more unbiased way produced clusters for patients with abundant T cells, B cells, monocytes/NK cells as seen in Fig. 1C (Supplementary Fig. 1D). The visualization of tSNE plots of T cells from these 4 groups appeared substantially different (Fig. 1D). Patients in G1 tended to have decreased CD4^+^ or increased CD8^+^ T cells, respectively, suggesting an unbalanced CD4/CD8 ratio. As shown in Fig. 1E, G1 had a significantly lower number of naïve (CD45RA^+^CCR7^+^, T_N_) and central memory (CD45RA^-^CCR7^+^, T_CM_) cells and a higher number of terminally differentiated (CD45RA^+^CCR7^-^, T_EMRA_) cells, suggesting a more inflammatory-like TME in G1. The number of T_CM_ and effector memory (CD45RA^-^CCR7^−^, T_EM_) cells was increased and decreased in G2, respectively. G3 showed an increased number of T_EM_ cells when compared to G1. In addition, the numbers of CD4^+^ and CD8^+^ cells that had downregulated costimulatory receptors (CD5, CD7, CD27, and CD28) were significantly higher in G1 than other groups (Fig. 1F). G3 had an increased representation of T_FH_ cells (PD-1^high^ICOS^high^) (Fig. 1G), consistent with the role of T_FH_ cells in promoting B-cell growth. The expression levels of surface markers that were significant among patient groups are summarized in Supplementary Fig. 1E. The statistical significance of surface markers and subsets among these four groups were corrected using Bonferroni post-test analysis.

We then determined T-cell phenotypic profiles (clusters) among patient groups using CITRUS analysis. As shown in Fig. 1H(i), T-cell phenotypic profile was substantially different in G1 compared to other groups, as more clusters emerged when G1 was compared to each of other three groups than when G2, G3 and G4 were compared to each other. T-cell clusters bearing KLRG1, CD57, PD-1^low^ or T_EMRA_ were significantly more abundant in G1 when compared to either G2, G3 or G4 (Fig. 1H(ii)). In contrast, T-cell clusters with phenotypes expressing CD127, CD45RA, CCR7 or PD-1^high^ were less abundant in G1 when compared to other groups. When compared to G3 or G4, 2 classes of T cell clusters, CD127^+^KLRG1^−^ (MPECs, memory precursor effector cells) or CD57^+^PD-1^high^ (CD57^+^ T_FH_), all from CD4^+^ T cells, were significantly more abundant in G2. Clusters with a phenotype of CD8^+^ SLECs (short-lived effector cells, KLRG1^+^CD127^-^) were upregulated in G3 when compared to G4 (Fig. 1H(iii)). The bootstrap analysis shown in Supplementary Fig. 1D revealed that G2 cluster was highly stable, verifying the findings related to G2 in this cohort. In contrast, G1 cluster was the least stable among these four groups, meaning that a large sample size is needed to verify the results related to G1. Of note, tissue from G1 was dominantly spleen tissue, while lymph nodes or other tissues were spread out across the G2–G4. RNA-seq analysis showed that the overall transcriptome of G1 was not significant different from the other groups (data not shown), suggesting that the biology rather than the tissue site contributes to varied T-cell phenotypes between G1 and other groups.

### Differentiation stage determines clinical relevance of T cells in FL

**Fig. 2:**
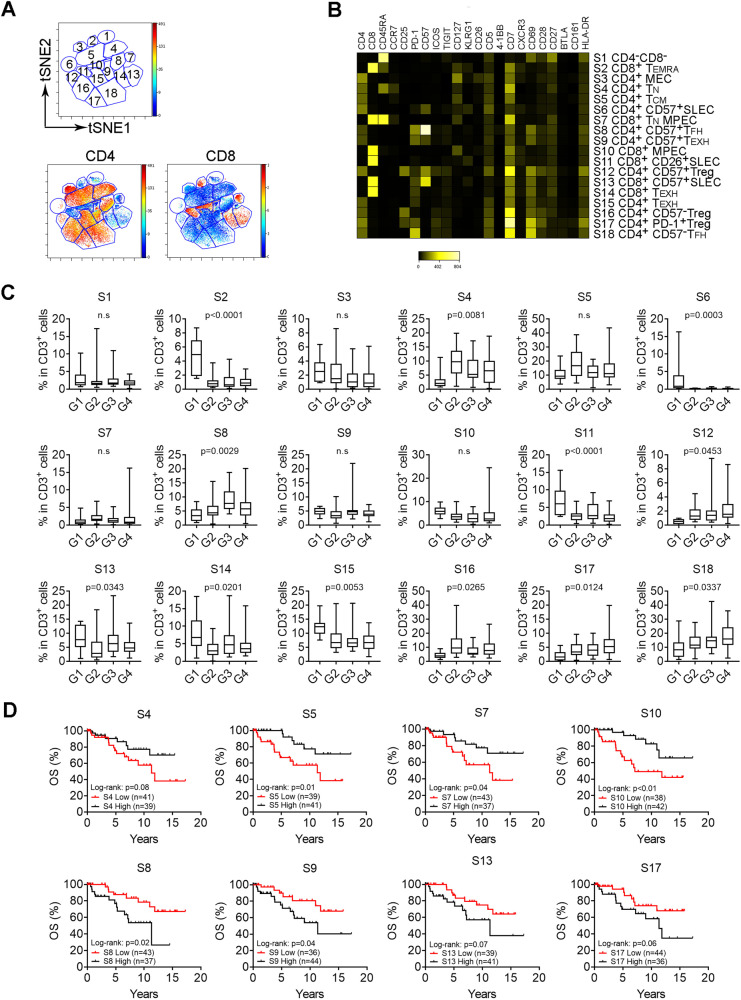
Differentiation stage determines clinical relevance of T cells in FL. **A** The tSNE plots of CD3^+^ T cells from a representative FL biopsy specimen. Subsets (S) circled with a number were identified by manual gating. **B** Heatmap showing median intensity of each marker for subsets identified above. Each subset was annotated based on the phenotype. **C** Graphs showing percentage of 18 subsets of T cells in each patient group. The p value was calculated using a one-way ANOVA analysis and indicates a difference between each group and the other groups. Of note, for S5, the Student *T* test showed that the *p* value was statistically significant when comparing G2 to either G1 (*p* = 0.048) or G3 (*p* = 0.034). **D** Kaplan–Meier curves of selected subsets for overall survival of FL patients (*n* = 80) using median number as cutoff point.

Next, we dissected the T cell compartment and manually and arbitrarily identified 18 T-cell subsets (S1–S18) using tSNE plots based on cell surface markers in FL (Fig. 2A, Supplementary Fig. 2). Of six CD8^+^ subsets, S7 was defined as naïve cells (T_N_, CD45RA^+^CCR7^+^) and five subsets were memory cells (CD45RA^−^) that included memory precursor cells (S10, MPEC, CD127^+^KLRG1^−^), short-lived effector cells (CD26^+^ S11/CD57^+^ S13, SLEC, KLRG1^+^CD127^−^), exhausted cells (S14, T_EXH_, PD-1^low^), and terminally differentiated cells (S2, T_EMRA_ CD45RA^+^CCR7^−^). Of 11 CD4^+^ subsets, one subset was naïve and 10 were memory cells with T_reg_, T_FH_, T_EXH_, T_CM_ cells being the major memory subsets in FL. For T_reg_ cells, expression of PD-1 and CD57 separated T_reg_ cells to 3 subsets with S12, S16 and S17 being CD57^+^, CD26^+^ and PD-1^+^, respectively (Fig. 2B, Supplementary Fig. 2).

The frequency of these subsets varied in patient groups identified above. As shown in Fig. 2C, memory CD8 subsets including MPEC (S10), SLEC (S11/S13), T_EXH_ (S14) and T_EMRA_ (S2) were significantly upregulated in G1 when compared to other groups. Subsets with early differentiation stage such as T_N_ (S4), T_CM_ (S5) were significantly upregulated in G2. CD57^−^ T_reg_ cells (S16) were also found to be increased in G2. Subsets of CD57^+^ (S8) and CD57^−^ T_FH_ (S18) were highly represented in G3 and G4, respectively. These results further support that T-cell phenotype varies in patients with distinct TMEs.

Dissection of the phenotypes allowed us to identify favorable or unfavorable T cell subsets based on their impact on patient outcomes. As shown in Fig. 2D and Supplementary Fig. 2B, S4, S5, S7 and S10 were associated with a favorable overall (OS) and time to next treatment (TTNT) while S8, S9, S13 and S17 correlated with an unfavorable survival. The frequency distribution plots of these 8 subsets are listed in Supplementary Fig. 2D. Consistent with this finding, Cox proportional hazard regression analysis showed that the hazard ratios (HR) were 0.59 (S4), 0.33 (S5), 0.65 (S7), 0.34 (S10), 2.42 (S8), 2.12 (S9), 2.01 (S13) and 1.87 (S17). As a control, the HR was 3.47 for age older than 60 years, a well-known prognostic parameter. Of note, subsets S4 (T_N_), S5 (T_CM_), S7 (T_N_) and S10 (MEPC) could be considered as cells with early-stage differentiation. In contrast, subsets S8 (CD57^+^T_FH_), S9 (T_EXH_), S13 (SLEC) and S17 (PD-1^+^T_reg_) were memory cells with later-stage of differentiation. Overall, increased numbers of CD45RA^+^ and CCR7^+^ T cells, representing T_N_ and T_CM_, correlated with a favorable survival (Supplementary Fig. 2C). Supporting this finding, CITRUS analysis revealed that clusters bearing a T_CM_ phenotype (CD45RA^-^CCR7^+^CD127^+^) were significantly more abundant in patients with early-stage (I–II) when compared to advanced-stage (III–IV, Supplementary Fig. 3A). Taken together, we defined T cells in early or late stages of differentiation as favorable or unfavorable cells, respectively.

We next determined whether the subsets with differing clinical significance were also phenotypically different. As shown in Supplementary Fig. 3B, favorable subsets (S4, S5, S7, and S10) exhibited markedly different phenotype from unfavorable subsets (S8, S9, S13, and S17). The numbers of cells expressing CD45RA, CCR7, CD127 and CD26 were significantly higher in S4, S5, S7 and S10 than that in S8, S9, S13 and S17. In contrast, surface markers including PD-1^low^, TIM-3, TIGIT, BTLA, ICOS, KLRG1, CD57, CD69, and HLA-DR were highly expressed in unfavorable subsets rather than favorable subsets.

### CCR6 expression defines B cells as a population with early differentiation stage that correlates with greater expression of costimulatory receptors on T cells

**Fig. 3:**
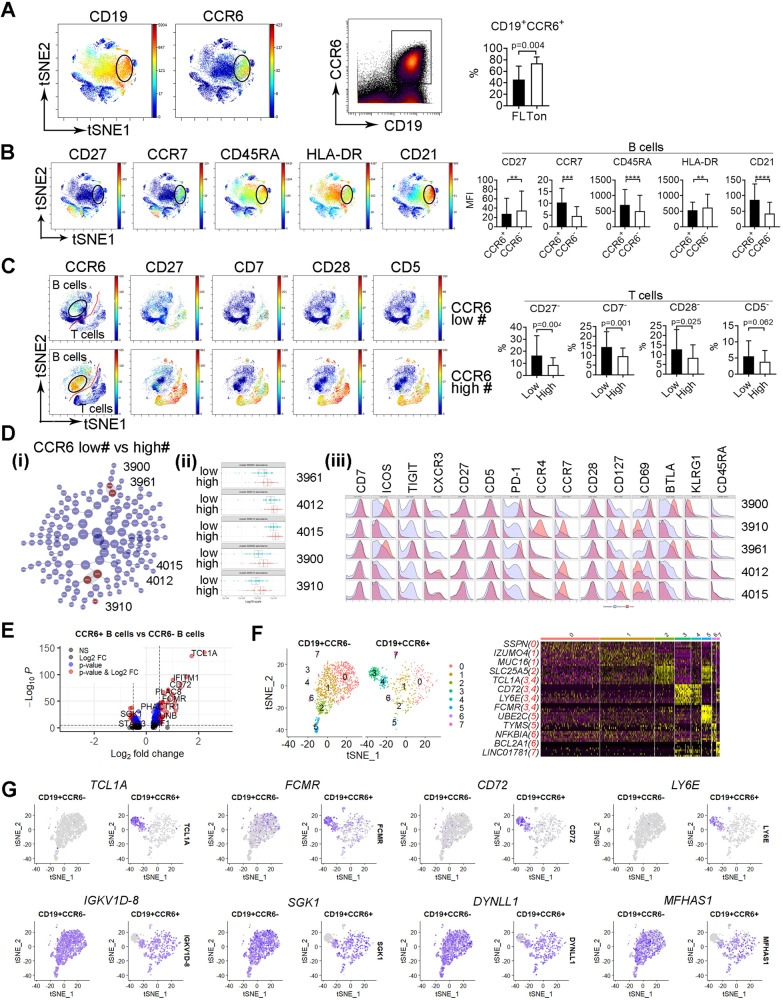
CCR6 expression defines B cells as a population with early differentiation stage that correlates with decreased deficiency of costimulatory receptors in T cells. **A** The tSNE and dot plots from a representative FL biopsy specimen showing expression of CD19 and CCR6 in CD45^+^ cells. Graphs showing percentage of CD19^+^CCCR6^+^ cells from 4 tonsil tissue and 82 FL specimens. **B** The tSNE plots of CD45^+^ cells showing expression of CD27, CCR7, CD45RA, HLA-DR, and CD21 from a representative specimen. Graphs showing mean fluorescent intensity (MFI) of surface markers above in CD19^+^CCR6^+^ and CD19^+^CCR6^−^ cells. **C** The tSNE plots of CD45^+^ cells showing expression of CCR6, CD27, CD7, CD28 and CD5 from patients with low or high number of CCR6^+^ cells. Graphs showing percentage of surface markers above in patients with low or high number of CCR6^+^ cells. **D** CITRUS plot showing clustering result from FL patients between patients with low or high number of CCR6^+^ cells. Circles in red represent clusters that differed between groups and number in circles indicates a cluster ID (i). Histogram plots showing expression of selected markers by cells from clusters overlaid on background staining (ii). Graph showing quantitative results of abundance from clusters (iii). **E** Volcano plots showing genes (dots) of upregulation (red), downregulation (blue) or no change (black) from CITE-seq analysis based on Log2 FC and adjusted *p* value. *P* < 0.05 indicates statistically significant. **F** The tSNE plot showing clusters from CD19^+^CCR6^−^ and CD19^+^CCR6^+^ cells based on gene expression profile from CITE-seq analysis. Heatmap showing gene expression by clusters from the tSNE plot. Representative genes were listed with cluster number in red. **G** The tSNE plots showing expression of genes TCL1A, FCMR, CD72, LY6, IGKV1D-8, SGK1, DYNLL1, and MFHAS1 from CD19^+^CCR6^−^ and CD19^+^CCR6^+^ cells in FL biopsy specimens.

We found that CCR6 expression distinguished CD19^+^ cells into subsets that are associated with distinct immune phenotypes. As shown in Fig. 3A, CCR6 was expressed on a subset of CD19^+^ B cells and CCR6^+^ cells accounted for ~45.3% of the total B cells. The frequency of CD19^+^CCR6^+^ cells was significantly lower in FL than control tonsil tissue. When compared to CCR6^-^ B cells, CCR6^+^ B cells exhibited significantly lower expression of CD27 and HLA-DR, as well as significantly higher expression of CCR7 and CD45RA (Fig. 3B). This expression profile indicated a phenotype of early differentiation stage for CD19^+^CCR6^+^ cells. Of note, CD19^+^CCR6^+^ cells were highly represented in patient group 2 (G2, Supplementary Fig. 4A), which is consistent with the observation that the number of memory B cells was significantly lower in G2 (Supplementary Fig. 4B).

Next, we explored whether the T-cell phenotype varied in patients with high or low numbers of CD19^+^CCR6^+^ cells. As shown in Fig. 3C, the intratumoral T cell phenotype in patients with more CCR6^+^ B cells displayed increased expression of co-stimulatory receptors (CD5, CD7, CD27, and CD28). The numbers of T cells losing expression of these costimulatory receptors were significantly lower in patients with more CCR6^+^ B cells than those with less CCR6^+^ B cells. Further analysis of T cells using CITRUS revealed that 5 clusters were significantly more abundant in patients with more CCR6^+^ B cells than those with less CCR6^+^ cells (Fig. 3Di-ii). Clusters 3900 and 3961 exhibited a phenotype of T_FH_ cells (PD-1^high^TIGIT^+^ICOS^+^BTLA^+^). Clusters 4012, 4015 and 3910 displayed a phenotype of T_CM_ cells (CD45RA^−^CCR7^+^) with 4012/4015 and 3910 being CXCR3^+^ and CCR4^+^, respectively (Fig. 3Diii). These 5 clusters had high expression of CD5, CD7, CD27 and CD28, consistent with the findings in Fig. 3C.

Gene expression profiling of CCR6^+^ cells using CITEseq showed a different gene expression profile when compared to CCR6^−^ cells (Supplementary Fig. 4C, D, Fig. 3E). Clustering analysis identified two distinct clusters (3 and 4) that were present in CCR6^+^ and not in CCR6^-^ B cells, with unique gene expression profiles (Fig. 3F). Cells from these two clusters expressed differentiation-related genes including *TCLIA*, a gene whose protein is strictly expressed on early B cells, *FCMR*, an IgM Fc receptor affecting pre-B and immature B cell differentiation, and *CD72*, a coreceptor of B cell receptor and a marker for early-stage B cells (Fig. 3F, G). This gene profile supported the observation that CD19^+^CCR6^+^ cells have an early differentiation stage phenotype.

### T cell phenotype is associated with histological grade in FL

**Fig. 4:**
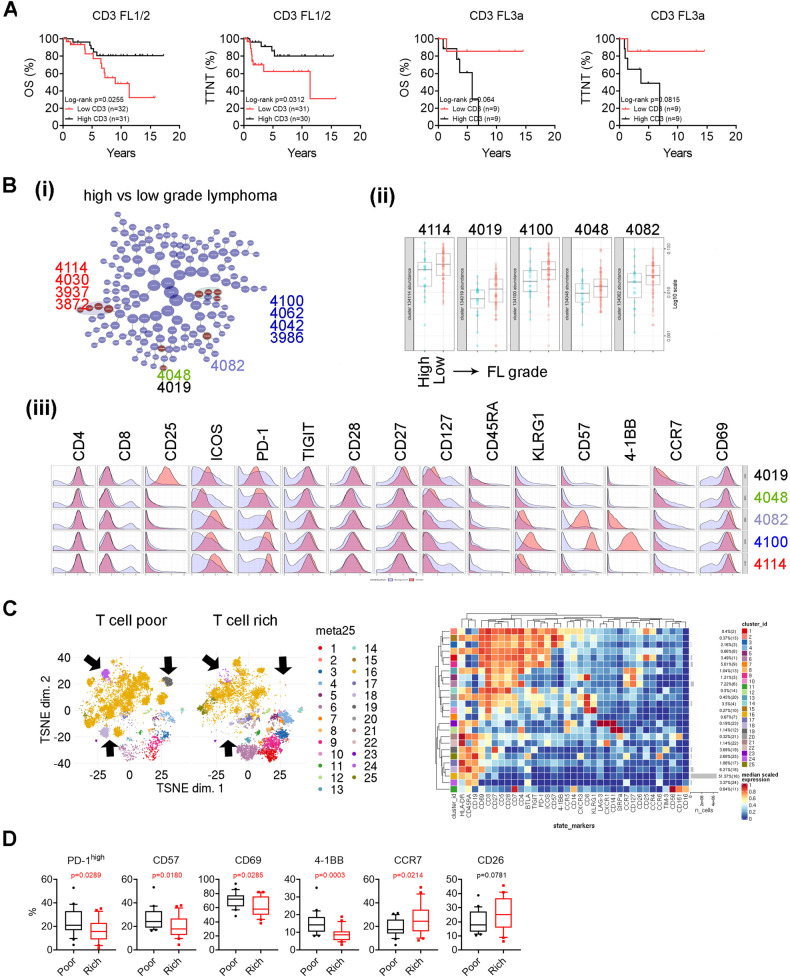
T cell phenotype is associated with histological grade in FL. **A** Kaplan–Meier curves of CD3 + T cells for OS and EFS in FL1/2 (*n* = 62) or FL3a (*n* = 20) patients using median number as cutoff point. **B** CITRUS plot showing clustering result from FL patients between patients with low- or high-grade lymphoma. Circles in red represent clusters that differed between groups and number in circles indicates a cluster ID (i). Graph showing quantitative results of abundance from clusters (ii). Histogram plots showing expression of selected markers by cells from clusters overlaid on background staining (iii). **C** The tSNE plots of CD45^+^ cells representing patients with either poor or rich in T cell number. Arrows to point to clusters that differed between patient groups. The heatmap (right) showing the phenotype and abundance (%) of each cluster. **D** Graphs showing percentage of PD-1^high^, CD57^+^, CD69^+^, CCR7^+^ and CD26^+^ cells in patients with either poor or rich in T cell number.

It has been demonstrated that increased numbers of intratumoral T cells are associated with a better survival. We, however, did not observe a similar clinical impact as T-cell numbers were not associated with patient survival in the entire cohort (Supplementary Fig. 5A). However, when we analyzed patients with FL1-2, we found that increased T cells (CD4^+^ and CD8^+^) significantly correlated with a favorable survival (Fig. 4A). We did not see a similar correlation with outcome in patients with FL3. Consistent with this observation, increased numbers of T cells also positively correlated with increased numbers of patients with younger age, early disease stage, no B-symptoms and those who achieved EFS24 in patient with FL1-2, but this effect was not seen in patients with FL3 (Supplementary Table 3).

We performed CITRUS to identify T-cell clusters that differed between these two groups. As shown in Fig. 4B, 11 clusters belonging to 5 classes were identified and significantly differed between patients with higher or lower grade lymphoma (i). These clusters all came from CD4^+^ cells and were highly represented in patients with lower grade lymphomas (ii). Clusters 4114 class including 4030, 3937 and 3872 expressed PD-1^high^, TIGIT, ICOS and lacked expression of CD57 and KLRG1 (iii). Clusters 4100 class including 4062, 4042, and 3986 as well as cluster 4082 also expressed PD-1^high^, TIGIT, ICOS, but had expression of CD57, 4-1BB and KLRG1. Cluster 4082 expressed lower level of CD57, 4-1BB, and KLRG1 than cluster class 4100. Cluster 4019 was CD25^+^ with PD-1^low^. These results indicate that T-cell phenotype varies in patients with low-grade FL and high-grade.

Given that the number of T cells significantly correlated with patient outcome in FL1-2, we wanted to explore whether patients with higher or lower T cell numbers exhibit distinct T-cell phenotypes. To do this, we grouped patients with T cell numbers in the top or bottom 25% as rich or poor (Supplementary Fig. 5B). Overall, phenotype in TME varied between patients with T cell rich and poor indicated by both clustering and multidimensional scaling (MDS) analysis (Fig. 4C, Supplementary Fig. 5C). As shown in Supplementary Fig. 5C, patients with T-cell rich or poor exhibited a well-separated scaling profile. Clustering analysis revealed that some clusters more abundant in patients with T-cell rich were less abundant or even lacked in patients with T-cell poor group and vice versa (Fig. 4C). In regard to T-cell phenotype, patients who were T-cell poor showed increased numbers of T cells expressing PD-1^high^, CD57, CD69 and 4-1BB, suggesting a phenotype of T_FH_ cells with terminally differentiated stage. Patients with a T-cell rich phenotype tended to have a T-cells in earlier stages of differentiation indicated by increased expression of CCR7 (Fig. 4D). Using CITRUS analysis, we identified 10 clusters that were significantly abundant in patients with a T-cell poor TME when compared to those with a T-cell rich TME (Supplementary Fig. 5D). Cluster 3954 was from CD8^+^ T cells and expressed KLRG1, CXCR3 and PD-1^low^, suggesting a phenotype of exhaustion. Clusters 3912 and 3934 were PD-1^high^, TIGIT^+^, and CD7^-^ with cluster 3912 and 3934 being with CD57^+^ and CD57^−^, respectively. Cluster 3962 exhibited a phenotype of T_EMRA_ (CD45RA^+^CCR7^-^CD57^+^TIM-3^+^). All other 6 clusters shared a similar phenotype of terminally differentiated T_FH_ cells (PD-1^high^TIGIT^+^CD7^+^CD57^+^). Of note, we observed a similar result when grouping patients using the median number of T-cells as a cutoff (Supplementary Fig. 5E).

### CD57^+^ T_FH_ cells are highly represented in patients with disease progression and correlate with inferior patient outcomes in FL

**Fig. 5:**
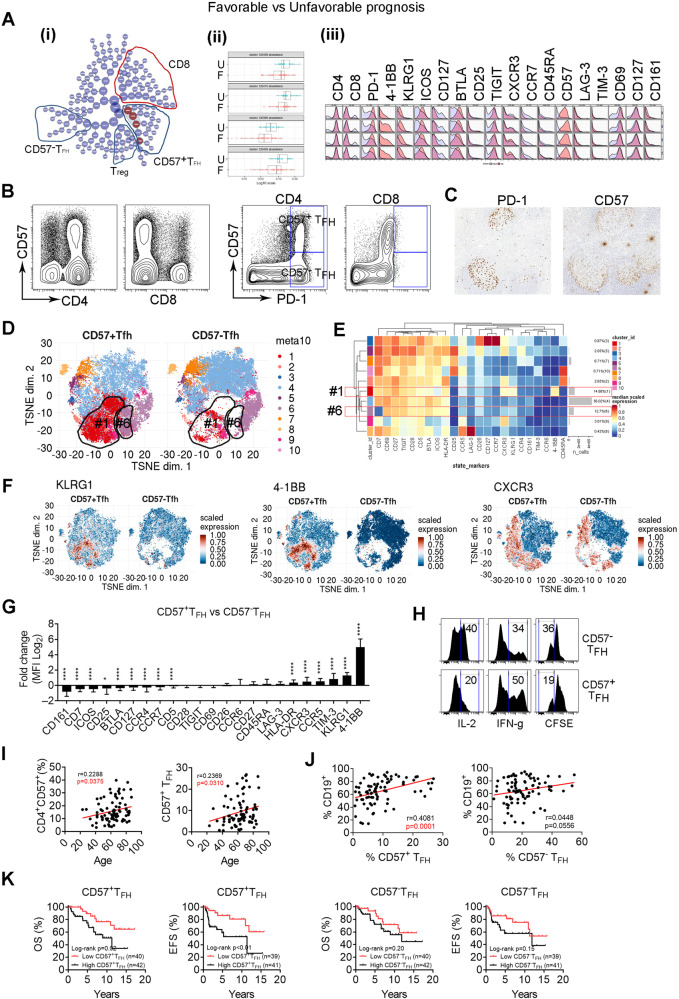
CD57^+^ T_FH_ cells are highly represented in patients with disease progression and correlate with inferior patient outcomes in FL. **A** CITRUS plot showing clustering result from FL patients between patients with CR/PR (favorable) and SD/Prog (unfavorable). Circles in red represent clusters that differed between groups (i). Graph showing quantitative results of abundance from clusters (ii). Histogram plots showing expression of selected markers by cells from clusters overlaid on background staining (iii). **B** Dot and tSNE (below) plots showing CD57 expression as well as coexpression of CD57 and PD-1 on CD4^+^ or CD8^+^ T cells. **C** Immunohistochemistry images showing PD-1 and CD57 staining on a tissue section from a FL patient, *n* = 2. Strong staining of PD-1 and CD57 were seen on cells within follicles. **D** The tSNE plots showing clusters from CD57^+^ and CD57^−^ T_FH_ cells based on surface marker expression profile from CyTOF analysis. Circled were clusters 1 and 6 to show difference between CD57^+^ and CD57^−^ T_FH_ cells. **E** Heatmap showing surface marker expression by clusters identified from Fig. 5d. The right column shows the percentage of each cluster. Cluster #1 and #6 were highlighted. **F** The tSNE plots showing expression of surface markers KLRG1, 4-1BB and CXCR3 from CD57^+^ T_FH_ and CD57^−^ T_FH_ cells. Graphs showing the percentage of CD57^+^ cells in CD4^+^ and CD8^+^ T cells. (**G**) Graph showing fold change (log2) of surface marker expression in CD57^+^ versus CD57− T_FH_ cells. **H** Histograms showing expression of IL-2, IFN-γ and CFSE^dim^ on CD57^+^ or CD57^−^ T_FH_ cells measured by flow cytometry, *n* = 3. **I** Scatter plots showing correlation between patient age and percentage of CD4^+^CD57^+^ or CD57^+^ T_FH_ cells. **J** Scatter plots showing correlation between percentage of CD19^+^ cells and CD57^+^ or CD57^−^ T_FH_ cells. **K** Kaplan–Meier curves for OS and EFS of FL patients (*n* = 80) by the number of CD57^+^ or CD57^−^ T_FH_ cells using median number as cutoff point.

Response to treatment in FL patients varies with good (complete (CR) or partial remission (PR)) and poor response (stable (SD) or progressive disease (PD)). To explore the difference of T-cell phenotype between patients with varied responses, we performed CITRUS analysis and found that 4 clusters were significantly different between patients with good (CR/PR) vs poor response (SD/PD). The abundance of these clusters was higher in poor response group than those with good response (Fig. 5A(i-ii)). Applying this analysis to patients with CR (representing the best response) vs PD (representing the worst response) showed a similar result (Supplementary Fig. 6A). These clusters exhibited a phenotype of CD4^+^, PD-1^hi^, ICOS^+^ and TIGIT^+^ cells, suggesting of T_FH_ cells. Cells from this cluster also expressed CD57 (Fig. 5A(iii)). Of note, we observed that there were clusters that were also CD4^+^, PD-1^hi^, ICOS^+^, TIGIT^+^, but CD57^−^ and showed no significant difference in prevalence between these two patient groups (Fig. 5A and Supplementary Fig. 6B). These results indicate that there are two subsets of T_FH_ cells: CD57^+^ and CD57^−^ T_FH_ cells. Similar results were seen in patients who achieved or failed EFS24 (Supplementary Fig. 6C), another clinical parameter indicating good or poor response.

This finding led us to explore CD57^+^ and CD57^−^ T_FH_ cells in FL. As shown in Supplementary Fig. 6D, CD57 was expressed on CD56^+^ NK cells from peripheral blood (PB), but not from lymph node (LN). While both CD19^+^ and CD14^+^ cells lacked CD57 expression, T cells from both PB and LN expressed CD57. Interestingly, while its expression can be seen on CD8^+^ T cells from PB and LN, CD57 was only expressed on CD4^+^ T cells from LN (Supplementary Fig. 6D). CD57^+^ cells accounted for ~17.4% (*n* = 82) of CD4^+^ and 29.6% (*n* = 82) of CD8^+^ cells, respectively (Supplementary Fig. 6E). While it was expressed on PD-1^low^ CD8^+^ T cells, CD57 was mainly expressed on CD4^+^ PD-1^high^ T cells that also expressed TIGIT and ICOS and resided within follicles – all of which are hallmarks of T_FH_ cells (Fig. 5B, C, Supplementary Fig. 6B). This expression pattern differentiated intratumoral CD4^+^ T_FH_ cells into CD57^+^ and CD57^−^ T_FH_ cells. These two subsets of T_FH_ cells exhibited different phenotypes based on a multidimensional scaling (MDS) plot (Supplementary Fig. 6F) in which CD57^+^ or CD57^−^ T_FH_ cells from patients clustered separate from each other. The tSNE plot (Fig. 5D, E) showed that two clusters (#1 and #6) were expanded in CD57^+^ T_FH_ subset when compared to CD57^−^ T_FH_. Cells from the cluster #1 expressed KLRG1 (a receptor expressed on highly differentiated end-stage cells or senescent cells), 4-1BB and CXCR3 (receptors that are expressed on antigen-experienced T cells) (Fig. 5F) and the cluster #6 included cells that lacked CD7 expression (a differentiation marker that plays an important role in T-cell and T/B interactions during early lymphoid development and is shed from the cell surface in later stage of differentiation). A comparison of surface marker expression profile between CD57^+^ and CD57^−^ T_FH_ cells as well as CD57^+^ and CD57^−^ CD8^+^ T cells is shown in Fig. 5G and Supplementary Fig. 6G. These phenotypical results suggest that CD57^+^ T_FH_ cells are late-stage cells.

Functionally, CD57^+^ T_FH_ cells displayed reduced capacity of IL-2 production and proliferation, but increased IFN-γ secretion when compared to CD57^−^ T_FH_ cells, a typical functional feature for late-stage memory T cells (Fig. 5H). We observed that CD57^+^ T_FH_ frequency positively correlated with advanced age (Fig. 5I) as well as the number of CD19^+^ lymphoma cells in FL (Fig. 5J), consistent with the finding that CD57^+^ T_FH_ cells were highly represented in the CD19^+^ B cell enriched patient group (G3). Clinically, CD57^+^ T_FH_ cells correlated with an inferior TTNT or OS (Fig. 5K), suggesting a detrimental effect on patient outcomes. To validate whether CD57^+^ T_FH_ cells are a risk factor independent of treatment response (favorable vs unfavorable), we performed a multivariate Cox proportional hazard regression analysis. The HR and 95% CI were 2.73 (95% CI: 1.1–7.7) for CD57^+^ T_FH_ and 0.88 (95% CI: 0.33–2.189) for treatment response. As a control, the HR was 1.42 (95% CI: 0.60–3.52) for CD57^−^ T_FH_ and 1.16 (95% CI: 0.46–2.77) for treatment response. These results confirm that increased CD57^+^ T_FH_ cells are a strong risk factor for patient outcome independent from treatment response and should be validated in an independent cohort.

### CD57^+^ T_FH_ cells are transcriptionally different from CD57^−^ T_FH_ cells and expression of CXCL13 may contributes to T_FH_ cell-mediated recruitment of B cells into follicles

**Fig. 6:**
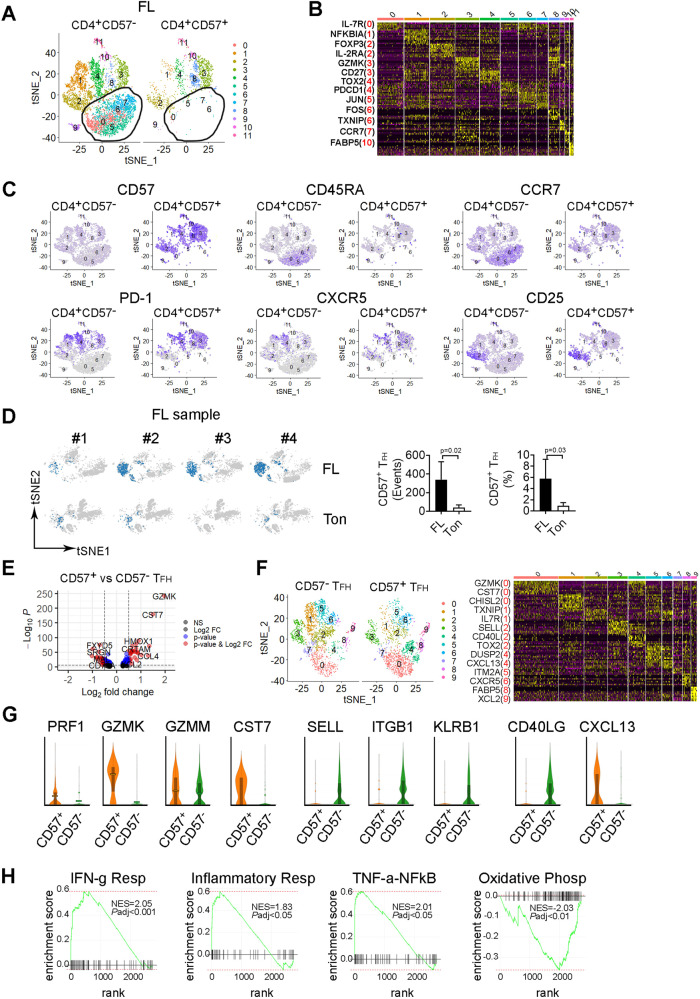
CD57^+^ T_FH_ cells are transcriptionally different from CD57^−^ T_FH_ cells and exhibit a gene expression profile of senescence. **A** The tSNE plot showing clusters from CD4^+^CD57^+^ and CD4^+^CD57^−^ cells based on gene expression profile from CITE-seq analysis. **B** Heatmap showing expression of upregulated genes (yellow) by clusters identified from Fig. 6A. Representative upregulated genes were listed from each cluster with the cluster number in red. **C** The tSNE plots showing expression of surface markers CD57, CD45RA, CCR7, PD-1, *CXCR5* (gene), and CD25 from CD4^+^CD57^+^ and CD4^+^CD57^−^ cells in FL biopsy specimens. **D** The tSNE plots of CITEseq analysis showing the content of CD57^+^ T_FH_ cells in 4 individual samples from FL and tonsil (Ton). Graphs showing number of events (cell counts) and percentage (in total mononuclear cells, MNC) of CD57^+^ T_FH_ cells in FL and Ton. **E** Volcano plots showing genes (dots) of upregulation (red), downregulation (blue) or no change (black) from CITE-seq analysis based on Log2 FC and adjusted *p* value. *P* < 0.05 indicates statistically significant. **F** The tSNE plot showing clusters from CD57^+^ and CD57^−^ T_FH_ cells based on gene expression profile from CITE-seq analysis. Heatmap showing gene expression by clusters from the tSNE plot. Representative genes were listed with cluster number in red. **G** Violin plots showing expression level of genes of PRF1, GZMK, GZMM and CST7, SELL, ITGB1 and KLRB1 as well as CD40LG and CXCL13 in CD57^+^ or CD57^−^ T_FH_ cells^.^ **H** GSEA plots showing enriched gene sets related to signaling pathways. NES normalized estimation score. NES with a positive or negative score indicates upregulation or downregulation, respectively.

To gain a better understanding of the transcriptome of CD57^+^ T_FH_ cells, we determined the gene expression profile of this subset using CITE-seq analysis from FL biopsy specimens different from the CyTOF cohort due to the unavailability of matched samples. While both RNA and protein markers can typically be used to identify specific subsets, the finding that expression of the CD57 gene B3GAT1 is extremely low in FL samples when compared to CD57 protein (Supplementary Fig. 7A) led us to use cell surface expression of CD57 for the gating strategy to identify and separate CD57^+^ and CD57^−^ T cells (Supplementary Fig. 7B). CD57 expression marked CD4^+^ T cells with advanced differentiation stages when compared to CD4^+^ lacking CD57 expression. As shown in Fig. 6A, clusters 0, 5, 6, and 7 were absent from the CD4^+^CD57^+^ T cell subset when compared to CD4^+^CD57^−^ T cells. These clusters represent cells with early-differentiation stage including naïve and central memory expressing CD45RA and CCR7 (Fig. 6B, C).

We next determined gene expression profile of CD57^+^ T_FH_ cells defined above and compared it to CD57^−^ T_FH_ cells. CD57^+^ T_FH_ cells were more abundant in FL when compared to tonsil, as both the number (cell count) and percentage of CD57^+^ T_FH_ cells were significantly higher in FL than control tonsil (Fig. 6D). Gene expression profile varied between CD57^+^ and CD57^−^ T_FH_ cells in FL (Supplementary Fig. 7C). A number of genes were significantly up- or downregulated in CD57^+^ T_FH_ when compared to CD57^−^ T_FH_ (Fig. 6E and Supplementary Table 4). Clustering analysis revealed unique profiles that were more or less abundant in CD57^+^ T_FH_ cells when compared to CD57^−^ T_FH_ (Fig. 6F). For example, cluster 0 featuring GZMK and CST7, and cluster 4 featuring DUSP2 and CXCL13 were upregulated in CD57^+^ T_FH_ cells. In contrast, cluster 1 featuring TXNIP and IL7R expression was downregulated in CD57^+^ T_FH_ cells when compared to CD57^−^ T_FH_ cells. Since CD57 is considered a hallmark of cytotoxic cells, as it is primarily expressed by NK and CD8^+^ T cells, it appeared that CD57 expression converted T_FH_ into cells with a cytotoxic phenotype. Genes encoding for granules and cytotoxic cell machinery, (*PRF1*, perforin; *GZMK*, granzyme K; *GZMM*, granzyme M) were highly expressed in CD57^+^ T_FH_ when compared to CD57^−^ T_FH_ cells (Fig. 6G). Supporting this, expression of *CST7*, the gene for cystatin F that has been shown to be a regulator of immune cell cytotoxicity [16], was significantly increased in CD57^+^ T_FH_ cells when compared to CD57^−^ T_FH_ cells (Fig. 6G). Of note, in addition to being a member of cytotoxic granules, it has been shown that GZMK is a hallmark of immune ageing [17, 18], supporting the augment that CD57^+^ T_FH_ are cells with late-stage differentiation. Adhesion molecule genes such as *SELL* (selectin L, CD62L), *ITGB1* (integrin subunit beta1), *KLRB1* (killer cell lectin receptor b1, CD161) were downregulated in CD57^+^ T_FH_ when compared to CD57^−^ T_FH_ (Fig. 6G). *CD40LG*, the gene encoding for CD40L that interacts with CD40 on B cells [19], was significantly lower in CD57^+^ T_FH_ than CD57^−^ T_FH_ cells, suggesting functional deterioration of CD57^+^ T_FH_ cells. We also observed that CD57^+^ T_FH_ cells expressed significantly higher levels of *CXCL13* than CD57^−^ T_FH_ cells. This observation may provide an explanation for the finding that the frequency of CD57^+^ T_FH_ cells was positively associated with the number of CD19^+^ cells, as studies showed that CXCL13-producing T_FH_ cells help recruit B cells to follicles [20–22]. We subsequently validated some differentially expressed genes by measuring protein expression using flow cytometry and CyTOF (Supplementary Fig. 7D, E). GSEA analysis revealed that gene sets related to IFN-γ response, inflammatory response and TNF-α signaling via NFκB were significantly enriched in CD57^+^ T_FH_ when compared to CD57^−^ T_FH_ cells (Fig. 6H).

### Spatial profiling of the TME in FL

**Fig. 7:**
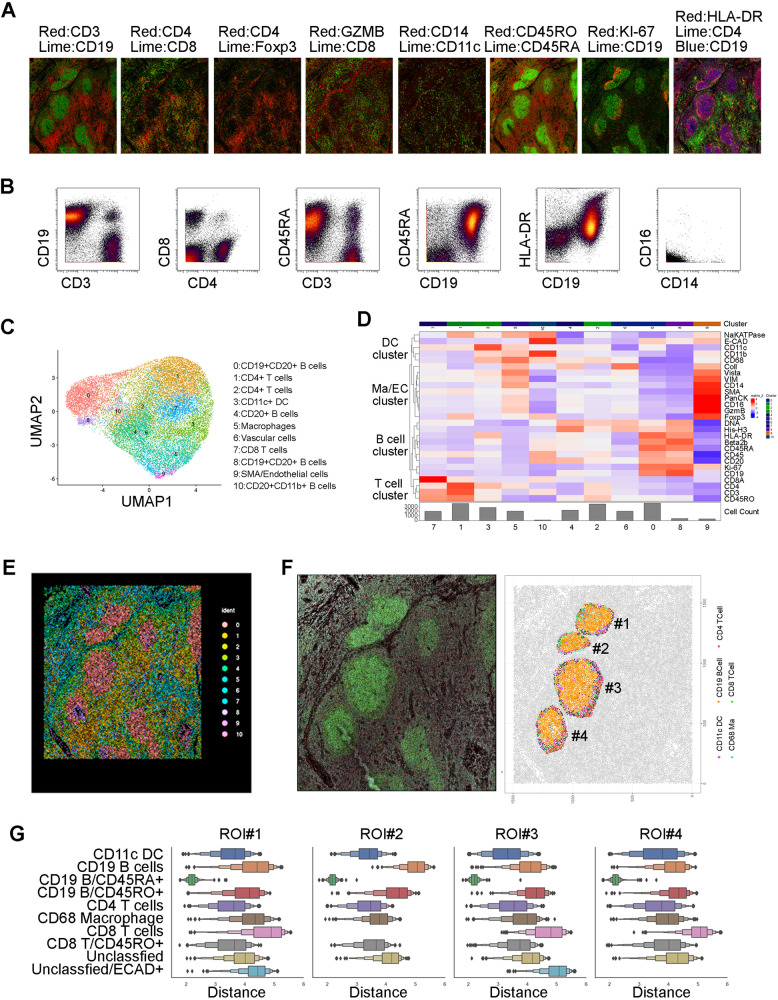
Spatial profiling of the TME in FL. **A** Stained images from multiplex imaging mass cytometry (MxIMC) using Hyperion from a region of interest (ROI) from a representative FL specimen. The whole slide was stained with 26 metal-conjugated antibodies and ROI were selected for Hyperion to scan. The images were viewed and merged using the MCD viewer (Fluidigm). **B** Dot plots showing surface marker expression by CyTOF from the same patient in (**A**). **C**, **D** The UMAP and Heatmap plots of cells from ROI of a representative FL patient. The data was extracted from segmentation process and analyzed with the R program (Seurat). 11 clusters were generated and annotated based on phenotype. **E** A spatial point plot with each color representing and indicating the origin of clusters from (**C**, **D**). The point is the X & Y coordinate of the centroid of each cell. This plot was analyzed and generated with the Seurat package. **F** CD19 staining (in Green) (left) alongside a spatial point plot produced using SciMap and color matching (right). **G** Distance plots, log of micron boxplots, using all the CD19^+^/CD45RA^+^ cells as the basis of pairwise comparisons by distance. Each boxplot was the distribution of that population’s distance away from all the CD19^+^/CD45RA^+^ cells, within each sub-regional annotation.

We then performed multiplex imaging mass cytometry (MxIMC) with Hyperion (Fluidigm) to gain a better understanding of the spatial location of cell populations in the TME in FL. Twenty-six antibodies (Supplementary Table 5) were used to stain lymph node tissue from 3 FL patients and included surface markers to define the structures of vessels (smooth muscle actin (SMA), vimentin (VIM), collagen I(Coll)), immune cell lineages (CD19, CD20, CD3, CD4, CD8, CD11c, CD11b, CD14, CD16), T-cell subsets (Foxp3, Granzyme B) and differentiation and activation states (CD45RA, CD45RO, Ki-67 and HLA-DR). As shown in a representative sample (Fig. 7A), the vast majority of CD19^+^ B and CD3^+^ T cells were reciprocally located in intra- and extra-follicular regions respectively, with some T (both CD4+ and CD8+) cells infiltrated into follicles. A small fraction of Foxp3^+^ cells were scattered in and outside follicles in a similar pattern in both FL and tonsil tissue. Cytotoxic granule granzyme B (GZMB) staining was abundant and only a small portion of GZMB^+^ cells were CD8^+^ T cells (yellow), given that GZMB can be expressed by a variety of cell types. CD11c^+^ cells are far more frequent than CD14+ cells in the TME. The staining of CD45RA and CD45RO was mutually exclusive on the cells between inside and outside follicles, as the vast majority of cells in the follicles were CD45RA^+^ while cells outside follicles were CD45RO^+^. A small portion of CD19^+^ cells around the follicles stained positive of Ki-67 while HLA-DR strongly co-stained with CD19^+^ cells in follicles. Utilizing CyTOF analysis, we validated some of these surface markers from this patient (Fig. 7B).

Next, we performed clustering analysis to identify cell subsets and 11 clusters were generated based on the phenotype (Fig. 7C). These 11 clusters represented 4 major cell clusters: namely a B cell cluster, T cell cluster, macrophage/endothelial cell cluster, and a DC cluster. The B cell cluster (CD19^+^ or CD20^+^), as expected, was the major cell population accounting for 4 clusters (0, 4, 8, and 10) with varied numbers. The T cell cluster accounted for clusters of CD4^+^ or CD8^+^ cells while CD11c^+^ cells were the major cells for DC cluster. Monocytes/macrophages (CD14^+^) and SMA/endothelial cells were clustered together and several markers such as Foxp3, GZMB were strongly stained in SMA/endothelial cells (Fig. 7D). The location of each cluster is shown in Fig. 7E. Next, we focused on the follicular areas and measured spatial proximity between lymphoma B cells and other immune cells. As shown in Fig. 7F, while B cells were the predominant cells in the follicles, other immune cells including CD11c DC, CD68 macrophages, both CD4^+^ and CD8^+^ T cells were seen around B cells and exhibited close proximity to B cells. CD4^+^ and CD8^+^ T cells were also seen in the follicles. Quantification analysis showed that different subsets were variably spatially associated with lymphoma B cells (Fig. 7G). Notably, we observed that CD4^+^ T cells had closer proximity to lymphoma B cells than CD8^+^ T cells or other immune cells. The proximity of CD4^+^ T cells to lymphoma B cells supports the importance of T cells in regulating the malignant cells.

## Discussion

The first question we addressed in the present study was whether patients with different TMEs show unique T-cell phenotypes. In previous work, we had begun to address this question. We had used immunohistochemistry on FL biopsy specimens, but the analysis was limited to a small number of cell surface molecules. We subsequently used CyTOF in a small number of patients, but this study included a larger cohort and allowed us to have statistical power to comprehensively explore the clinical relevance of intratumoral T-cell subsets in FL. Focusing on the major mononuclear cell lineages, we stratified patients into groups with different TMEs. Our results clearly showed that there is a difference in the T-cell phenotype in patients with various TMEs. Patients with TMEs rich in monocyte/macrophages/NK cells tended to have fewer T_N_, but more T_EMRA_ and T_EXH_ cells, which is consistent with observations that monocytes/macrophages are a marker for poor prognosis in FL [23, 24]. Patients enriched with T cells exhibited a T-cell phenotype skewed towards an early differentiation stage and patients with B-cell dominance tended to have significantly higher number of T_FH_ cells, further confirming a critical role of malignant B cells as well as the TME in modulating the T-cell phenotype [25, 26]. In patients with fewer T cells, a mechanism by which T-cell are excluded may be involved but this has yet to be explored in FL [27]. Recent data suggest that different B-cell NHL entities have characteristic quantitative patterns of T-cell infiltration [28], thereby supporting our finding that different TMEs in follicular lymphoma show unique T-cell phenotypes.

The second question we addressed was whether T cell differentiation status has differing clinical impact in FL. We observed that T subsets with distinct differentiation stage differentially correlated with patient outcome, which validated our previous finding in separate cohorts [15, 29]. We therefore defined T cells with early differentiation stage as favorable cells and memory T cells with late differentiation stage as unfavorable cells in FL. The findings that less-differentiated T cells exhibit enhanced self-renewal ability, increased persistence, and reduced inhibition by T_reg_ cells thereby resulting in superior immune responses against tumor cells [30–34], provide an explanation to our findings.

The third question we wanted to answer was whether there is a differential role of T cells in predicting outcome in histologically low- (FL1-2) or higher-grade (FL3) FL. Our results validate this with the finding that increased T cells significantly correlated with a favorable survival and other clinical parameters in low grade patients, but not in patients with higher grade FL. It has been shown that higher grade lymphoma, especially FL3b, is different from lower grade FL in its cytogenetic and immunohistochemical profile such as BCL2, BCL6, MYC and IRF4 [35, 36], which may contribute to the development of a different TME.

Whether patients with varied responses to treatment show a distinct T-cell phenotype is also unclear. By analyzing the T-cell phenotype in patient groups by treatment responses (CR, PR, SD and PD), we found that clusters with phenotype of CD57^+^T_FH_ cells was more abundant in patients with unfavorable responses (SD/PD/EFS24 failed) than favorable responses (CR/PR/EFS24 achieved). Our findings are consistent with a previous study showing that CD57^+^ T cells were associated with a significantly higher frequency of adverse clinical-biological manifestations such as “B” symptoms and bone marrow involvement [37].

CD57 was originally defined as a marker for NK [38] and CD8^+^ T cells [39] and subsequent studies observed that CD57 is also expressed on a T subset in germinal centers (GC) where T_FH_ cells reside [40–43]. It has been shown that CD57^+^ T cells were more efficient in GC B cell stimulation compared to CD57^−^ CD4 T cells [43], which is consistent with our finding that the number of CD57^+^ T_FH_ cells positively correlated with the number of B cells in FL. Our phenotypic analysis suggested that CD57^+^ T_FH_ cells are a population in a late differentiation stage, consistent with previous studies showing that CD57^+^ T cells are senescent cells [44, 45]. Transcriptomic profiling revealed that GZMK expression is significantly upregulated supports the contention that CD57^+^ T_FH_ cells are late-stage cells, as studies show that GZMK is a hallmark of immune ageing [17, 18]. Using single cell RNA sequencing, a recent study identified a major CD4^+^ subset expressing GZMK in the follicles in FL, a subset sharing a similar phenotype to CD57^+^ T_FH_ cells [46]. Also, upregulation of CXCL13 by CD57^+^ T_FH_ cells, but not CD57^−^ T_FH_ cells, may be a mechanism for the finding that the frequency of CD57^+^ T_FH_ cells was positively associated with the number of CD19^+^ B cells, in that studies have shown that CXCL13-producing T_FH_ cells help recruit B cells to follicles [20–22]. Taken together, our results identified CD57^+^ T_FH_ cells as a unique population that differed from CD57^−^ T_FH_ cells phenotypically and functionally, thereby differentially impacting patient outcome in FL. However, future validation in an independent cohort to confirm these findings is warranted.

## Methods

Further information about the methodology, including the statistical analyses, is included in Supplementary Methods.

### Reporting summary

Further information on research design is available in the Nature Research Reporting Summary linked to this article.

### Supplementary information

Supplementary materials

Reporting Summary

## Data Availability

The dataset generated during and/or analyzed during the current study are available from the corresponding author on reasonable request.
