# Supplementary material for: T-cell phenotype including CD57+ T follicular helper cells in the tumor microenvironment correlate with a poor outcome in follicular lymphoma
Source: Blood Cancer J. 2023 Aug 18;13(1):124. doi: 10.1038/s41408-023-00899-3 (PMC10435479; doi:10.1038/s41408-023-00899-3)
Supplement: Supplementary file 1 — Supplementary materials [file 41408_2023_899_MOESM1_ESM.docx]

**Methods**

*Patient samples*. Patients providing written informed consent were eligible for this study if they had a tissue biopsy that on pathologic review showed FL and adequate tissue to perform the experiments. Reactive spleen and lymph nodes from patients with hyperplasia as well as PBMCs and tonsils from healthy donors were used as controls. The use of human tissue samples for this study was approved by the Institutional Review Board of the Mayo Clinic/Mayo Foundation.

*Cell isolation and purification*. Fresh tumor biopsy specimens from patients with FL and control LNs were gently minced over a wire mesh screen to obtain a cell suspension. The cell suspension or peripheral blood from patients or healthy donors was centrifuged over Ficoll Hypaque at 500 x g for 20 minutes to isolate mononuclear cells. Cells were isolated using negative selection with microbeads (StemCell Technologies, Vancouver, Canada).

*Flow cytometry and intracellular staining*. For surface marker detection, 1x10^6^ cells were washed in phosphate-buffered saline (PBS) containing 0.5% bovine serum albumin and incubated with fluorochrome-conjugated antibodies (Abs) and analyzed on a flow cytometer. For intracellular staining, cells were stimulated with PMA/ion in the presence of protein transport inhibitor brefeldin A for 4 hours. After fixation and permeabilization, cells were stained with fluorochrome-conjugated Abs for cytokines and analyzed by flow cytometry. For apoptosis detection, cells were cultured with 2µL Dynabeads^@^ human T-activator CD3/CD28 in 96-well plate at 37°C in the presence of 5% CO_2_. On day 3, cells were stained with PI /annexin V-FITC and analyzed by flow cytometry.

*CFSE labeling and T cell proliferation assay*. Cells were resuspended at 1×10^6^/ml in PBS. A stock solution of CFSE (5 mM) was added to the cells for a final concentration of 5 μM. After 10 minutes at room temperature, cells were washed 3 times with 10 volumes of PBS containing 10% FBS. CFSE-labeled T cells were cultured with 2µL Dynabeads^@^ human T-activator CD3/CD28 in 96-well plate at 37°C in the presence of 5% CO_2_. Cells were harvested at day 3 or day 7 and analyzed on a flow cytometer.

*Immunohistochemistry.* Paraffin-embedded tissue was obtained from Mayo Clinic Tissue Registry and the tissue sections were deparaffinized in three changes of xylene. After cleared through graded ethanol series, endogenous peroxidase was quenched by incubation in 50% methanol/H_2_O_2_. After rinsing with tap water, all sections were pretreated 30 minutes with 50 mmol/L EDTA (pH 8) using a steamer and cooled for an additional 5 minutes. The staining was performed using anti-PD-1(abcam #52587, 1:50) or anti-CD57(Novus biologicals #NBP2-48366, prediluted). The sections were stained with hematoxylin and rinsed well in tap water. The slides were observed and images were captured with MOTIC Easyscan.

*CyTOF assay.* The CyTOF assay was performed according to the manufacturer’s instruction. Briefly, 3 × 10^6^ cells were stained with 5 µM Cell-IDTM Cisplatin (Fluidigm, San Francisco, CA) for 5min and quenched with MaxPar Cell Staining Buffer (Fluidigm) using 5 times the volume of the cell suspension. After centrifugation, cell suspensions (50 µl) were incubated with 5 µL of human Fc-receptor Blocking solution (Biolegend, San Diego, CA) for 10min and 50 µL of pre-mixed antibody cocktail for 30 min. For the staining antibody panel, please refer to this publication([1](#_ENREF_1)). After washing, cells were incubated with 1ml of cell intercalation solution (125nM MaxPar Intercalator-Ir into 1ml MaxPar Fix and Pem Buffer, Fluidigm) overnight at 4˚C. Cells were centrifuged with MaxPar Water and pelleted. The pelleted cells were suspended with EQ Calibration Beads (Fluidigm) and cell events were acquired by a CyTOF Helios instrument (Fluidigm).

*CyTOF data analysis.* The CyTOF data were analyzed using online software (Cytobank) as previously described ([2](#_ENREF_2),[3](#_ENREF_3)) and R script ([4](#_ENREF_4)). To minimize the batch effects, the Mayo Immune Monitoring Core that runs CyTOF assay establishes a standard protocol and takes every necessary step. These steps include: (1) Data are collected at an event rate no higher than 300 events/second; (2) All samples are stained by the same individual; (3) All samples are run on the same CyTOF instrument; (4) All samples are spiked with the same batch of EQ Four Element Calibration Beads (PN 201078;Standard BioTools); (5) Helios mass cytometer/CyTOF is tuned to the manufacturer’s specification prior to each batch of acquired samples; (6) Samples from each test group are included in each batch; (7) Antibody cocktails are pre-made prior to start of the experiment; (8) Equivalent numbers of cells are used and harvested for each sample. These steps minimize the variation of signal decay among samples. In addition, normalizing all the samples simultaneously also helps minimize the variation of signal decay among samples. During the analysis, we determine batch effects by observing whether the clusters overlap across batches and whether there are the discrete clusters that map back to a specific day of staining. For specific analysis purposes, we concatenated all sample files to one file. To generate flow files for comparison of distinct populations of interest, we split files by the population and downloaded them into individual files for each sample.

A tSNE plot was generated by the t-Distribution Stochastic Neighbor Embedding (tSNE) analysis that makes a pairwise comparison of cellular phenotypes to optimally plot similar cells close to each other and reduces multiple parameters into two dimensions (tSNE1 and tSNE2). For most analysis, we selected equal events for each sample. Channel (markers) selection was variable depending on cell populations to be clustered. We chose 3,000 iterations, perplexity of 30 or 50 and theta of 0.5 as standard tSNE parameters.

For CITRUS (cluster identification, characterization, and regression) analysis, models of significance analysis of microarrays (SAM) and nearest shrunken centroid (PAMR) were selected for association analysis. Abundance was chosen for the cluster characterization and the minimal cluster size was 0.5%. Patients were divided into two groups based on survival (patients dead or alive at last follow up). CITRUS analysis was performed on subsets and we used a number of surface markers to identify clusters that share a unique phenotype. Using correlative (SAM) or predictive (PAMR) methods, CITRUS analysis was able to identify the clusters (populations) that differ significantly between the patient groups by cell abundance (cell events).

To determine the stability of clusters, the bootstrap analysis was performed. Briefly, we performed an internal (leave-one-out) approach where we randomly removed 5 samples and reperformed the clustering. This was repeated 5 times. Sample assignments in clusters after each run were annotated and compared to determine cluster stability.

*CITE-seq assay.* CITE-seq was performed according to the manufacturer’s instructions (10x Genomics, USA). Cells (5x10^5^) were resuspended in 50μL staining buffer and incubated for 10 mins with an Fc receptor blocker (Human TruStain FcX, BioLegend, USA). Subsequently, cells were incubated with mixtures of 35 TotalSeq™-C antibodies (1μg per Ab per sample, the panel of antibodies is the same as the one in CyTOF that provided in Supplementary Table 2) for 30 min at 4 °C. Cells were washed three times in cell staining buffer, followed by centrifugation (350g 5 min at 4 °C). After the final wash, cells were resuspended at appropriate cell concentration (700-1200 cells/µL, viability>90%) in calcium and magnesium free 1×PBS (Corning, USA) containing 0.04% BSA (Thermo Fisher Scientific, USA) and run by 10x Genomics applications. The cells were first counted and measured for viability using the Vi-Cell XR Cell Viability Analyzer (Beckman-Coulter). The barcoded Gel Beads were thawed from -80C and the cDNA master mix was prepared according to the manufacturer’s instructions for Chromium Single Cell 5’ Library and Gel Bead Kit (10x Genomics). Based on the desired number of cells to be captured for each sample, a volume of live cells was mixed with the cDNA master mix. The cell suspension and master mix, thawed Gel Beads and partitioning oil were added to a Chromium Single Cell A chip. The filled chip was loaded into the Chromium Controller, where each sample was processed and the individual cells within the sample were partitioned into uniquely labeled GEMs (Gel Beads-In-Emulsion). The GEMs were collected from the chip and taken to the bench for reverse transcription, GEM dissolution, and cDNA clean-up. The full-length cDNA was amplified and separated by size selection. The resulting cDNA created a pool of uniquely barcoded molecules used to generate 5’ gene expression libraries (GEX). In addition, the supernatant from the cDNA clean-up step contained amplified DNA from cell surface protein feature barcodes. That DNA was further cleaned and used to create cell surface protein libraries. During library construction, standard Illumina sequencing primers and a unique i7 Sample index (10x Genomics) were added to each cDNA and DNA pool (creating gene expression and feature barcodes libraries respectively). All cDNA and DNA pools and resulting libraries were measured using Qubit High Sensitivity assays (Thermo Fisher Scientific) and Agilent Bioanalyzer High Sensitivity chips (Agilent).

Gene expression libraries (GEX) were sequenced at a minimum of 50,000 fragment reads per cell and feature barcodes libraries were sequenced at 5000 fragment reads per cell. Sequencing steps followed Illumina’s standard protocol using the Illumina cBot and HiSeq 3000/4000 PE Cluster Kit. For gene expression libraries, the flow cells were sequenced as 100x 2 paired end reads on an Illumina HiSeq 4000 using HiSeq 3000/4000 sequencing kit and HCS v3.3.52 collection software. For feature barcodes libraries, the flow cells were sequenced as 100x2 paired end reads on an Illumina HiSeq 4000. Base-calling was performed using Illumina’s RTA version 2.7.3.

*CITE-seq analysis.* The raw 10x CITE-seq data including mRNA and ADT libraries were processed using Cell Ranger v3.0.0 to produce gene and protein expression tables. After sequencing analysis, FASTQ files were created by the Cell Ranger v3.0.0 mkfastq pipeline (10× Genomics). The obtained FASTQ files were mapped to the human reference genome GRCh38. Cell Ranger count pipeline (v3.1.0) was used to perform demultiplexing, aligning reads, filtering analyses, using default parameters. The pipeline cellranger aggr was performed for aggregating outputs from several runs of cellranger count or cellranger multi pipeline. The quality-assured data from Cell Ranger output was recovered for secondary analysis of gene and ADT expression.

Single cell RNA-seq - Filtered gene count per cell matrices for each FL and tonsil sample from were independently pre-processed and filtered using Seurat R package. The total numbers of UMIs and genes for each cell were counted. The upper bound was calculated as mean plus two standard deviations (SD) and the lower bound as mean minus two SD for both the total UMIs and genes. Cells with total UMIs or genes outside of the upper and lower bounds were removed. Cells were removed if greater than 10% reads mapped to mitochondrial genes. Cells expressing less than 100 genes were removed from analysis. We also removed genes expressed in less than 5 cells. The filtered counts were normalized in Seurat v4 by a global-scaling normalization and log-transform method (LogNormalize) that normalizes the gene expression measurements for each cell by the total expression and multiplies it by a scale factor (10,000) and log-transforms the result. The top 2,000 highly variable genes (HVGs) were selected in each dataset with the function FindVariableGenes. The processed data and HVGs were used as input to perform batch correction using Seurat v3. The Uniform Manifold Approximation and Projection (UMAP) plots were generated from the batch-corrected low-dimensional embedding matrices. Before batch correction 8 scRNA-seq datasets showed strong batch effects in UMAP plots, clustering by group (FL and Tonsil) instead of by cell type. After Seurat v3 batch correction, separation between two groups were significantly reduced.

Single cell ADT: For single-cell antibody-derived tags (ADTs) in CITE-seq we normalized the ADT expression within a cell using the centered-log ratio (CLR) transform. In CLR transformation, sample vectors undergo a transformation based on the logarithm of the ratio between the individual elements and the geometric mean of the vector. We use the notation g(x) to indicate the geometric mean of the sample vector, x. Since these transformations apply to each sample vector independently, the presence of an outlier sample does not alter the transformation of the other samples:

$$clr\left( x \right)= [ln\frac{xi}{g\left( x \right)};\ldots;ln\frac{xD}{g\left( x \right)}]$$

We did not perform feature selection on ADT data and used all measured ADTs during dimensional reduction. Like scRNA the processed single cell ADT datasets for FL and tonsil samples were then integrated using Seurat v3 integration.

The RNA and ADT assays were independently scaled and the Principal Component Analysis (PCA) was performed. Using the Weighted Nearest Neighbor procedure implemented in Seurat v4, we calculated cell specific modality weights for scRNA and ADT and constructed a WNN graph based on top 20 PCA vectors from RNA assay and top 18 vectors from ADT assay. Cells were clustered using Seurat FindClusters function and Clustering was visualized in two-dimensional scatter plots (via UMAP). The cluster representing the T_FH_ cells were identified using the expression of ADT markers CD3^+^CD4^+^CD8^-^CD19^-^PD-1^high^. Differential gene expression analysis between CD57^+^ and CD57^-^ T_FH_ cells was completed using the Seurat FindMarkers function, employing a Wilcoxon rank-sum test. P value adjustment was performed using Bonferroni correction After differential gene expression profile was extracted, pathway analysis was performed using fast preranked gene set enrichment analysis (GSEA) package in R software to identify gene sets from the Molecular Signature Databases.

*Hyperion Panel Staining.* Hyperion staining was performed at the Pathology Research Core (Mayo Clinic, Rochester, MN). Slides were baked in a 60°C oven for 60 minutes. Slides were loaded into a Leica Bond RX stainer to perform dewaxing/baking, heat induced antigen retrieval and blocking steps. An Epitope Retrieval 2 solution (EDTA based, Leica) was used for 20 minutes. Slides were blocked in Superblock solution (Thermo Fisher) for 30 minutes, followed by a few washes in PBS-TB. Slides were removed from the stainer and placed in PBS-TB while preparing an antibody cocktail followed by manual staining. Metal-labeled antibodies (Supplementary Table 5) were either bought directly from Fluidigm or conjugated by the Immune Monitoring Core (IMC). Antibodies were spun down at 13,000 x g for two minutes prior to preparing the antibody cocktail, then diluted in PBS-TB according to Table 1 and incubated overnight at 4°C in a humidity chamber. Prior to adding the antibody cocktail to each slide, the tissue was encircled using a PAP pen. After overnight incubation, slides were washed three times for five minutes each in PBS-TB and incubated in Cell ID Intercalator-Ir (Fluidigm) at 1:400 for 30 minutes. Slides were washed in PBS-TB three times for five minutes each, followed by a rinse in Milli-Q water for five minutes. Slides were allowed to air dry and were sent to the IMC lab for imaging of region of interest (ROI). The merged images were viewed and generated using the MCD™ Viewer (Fluidigm).

*Imaging data analysis:* We have expanded upon the IMC workflow for pixel classification based segmentation from the Bodenmiller Group pipeline guidelines (5). This adaptation around select channels minimizes the required annotation effort for pixel classification without compromising performance. Pixel classification based segmentation combines the chosen channels of Hyperion datasets with annotations representing cellular & non-cellular components via training of our own model thus giving us higher precision and specificity in predicting cell masks in our own data. Once a model is confidently resolved, we return to the subset panel OME.TIFF of the entire capture area, and use Ilastik’s headless mode to predict pixel classes across the entire tissue. The product from this is a 3 channel prediction matrix, one channel per class, which then becomes the input to Cell Profiler pipeline (6). The CellProfiler pipeline takes those pixel probabilities and generates a nuclear mask via PrimaryObjectDetection which in turn feeds into the SecondaryObjectDetection. Thus, ensuring that every mask for one cellular component is guaranteed to have a partner. The output from CellProfiler is two corresponding labeled mask files. From here, we can reintegrate the cell segmentation masks with the full panel design by creating CellDetectionObjects in QuPath (7) where each mask resolves to a single geometric roi. These quantified cells now require an assignment of context, as the quantification values can still contain noisy data values, so we attempt to raise the signal by grouping cells into phenotypic categories. This classification can be done by supervised or unsupervised means. Obtaining upfront annotation can be tricky, so we start by leveraging the Seurat software via R programming language (8). This package is commonly used to generate an unsupervised cluster in a dimensionally reduced hypergeometric space. As such there are a number of considerations to account for in the parameterization of this approach, so first we perform feature selection to identify the most resolvable channels and iterate resolution values to identify the most stable clustering amount. This provides us with UMAP and tSNE graphs to identify unique cell populations [CD11C Dendritic, CD68 Macrophage, CD19 BCell, CD8 TCell, CD4 TCell, Unclassified, CD19 BCell/CD45RA^+^,CD19 BCell/CD45RO^+^,CD8 TCell/CD45RA^-^/CD45RO^+^, Unclassified | ECAD^+^]. Upon completion of assignment of phenotypic context we now have data that contains a two-dimensional spatial coordinate and biologically relevant definition(s).

The key spatial analysis is a permutation test, originally employed in Histocat manuscript (9), more recently available through the SciMap package (10). In this context, we are interested in whether cell-cell interactions are random, increased or decreased in one set of images compared to another. We define a radius of distance (typically 30-50 µm) to aggregated cell associations, permute the co-occurrences of cells within that distance and statistically calculate the likelihood of random or non-random occurrence. Interactions are intended to reveal cellular pressures of existence, colored in gray are statistically random occurrences. Red/higher values indicates empirical observations that are greater than random, and blue/lower values indicate observations that are less frequent than random.

*Statistical methods*. Statistical analysis was performed using the Student's t test. Significance was determined at p<0.05. For non-parametric data, a Mann-Whitney test or Wilcox matched-pairs rank-test was performed. Overall survival (OS) or time to next treatment (TTNT) was measured from the date of diagnosis until death from any cause or until the date of next treatment, respectively. OS or TTNT of all patients was estimated using the Kaplan-Meier method. The univariate associations between individual clinical features and survival were determined with the log-rank test. EFS24 (event-free survival at 24 months) achieved or failed was defined as patients who remained in remission or progressed within 24 months of diagnosis. We utilized the median number of each subset as a cutoff point to delineate patients with high or low content of each T subsets. For the data sets with multiple groups, we performed the Bonferroni post-hoc test to correct and validate the statistical significance. Statistical analysis was performed using software GraphPad Prism 8 and JMP 14.

1. Yang ZZ, Kim HJ, Wu H, Jalali S, Tang X, Krull JE*, et al.* TIGIT Expression Is Associated with T-cell Suppression and Exhaustion and Predicts Clinical Outcome and Anti-PD-1 Response in Follicular Lymphoma. Clin Cancer Res **2020**;26(19):5217-31 doi 10.1158/1078-0432.CCR-20-0558.

2. Kotecha N, Krutzik PO, Irish JM. Web-based analysis and publication of flow cytometry experiments. Curr Protoc Cytom **2010**;Chapter 10:Unit10 7 doi 10.1002/0471142956.cy1017s53.

3. Yang ZZ, Kim HJ, Villasboas JC, Price-Troska T, Jalali S, Wu H*, et al.* Mass Cytometry Analysis Reveals that Specific Intratumoral CD4(+) T Cell Subsets Correlate with Patient Survival in Follicular Lymphoma. Cell Rep **2019**;26(8):2178-93 e3 doi 10.1016/j.celrep.2019.01.085.

4. Nowicka M, Krieg C, Crowell HL, Weber LM, Hartmann FJ, Guglietta S*, et al.* CyTOF workflow: differential discovery in high-throughput high-dimensional cytometry datasets. F1000Res **2017**;6:748 doi 10.12688/f1000research.11622.3.

5. Jonas Windhager, Bernd Bodenmiller, Nils Eling (2020). An end-to-end workflow for multiplexed image processing and analysis. bioRxiv, doi: 10.1101/2021.11.12.468357. https://bodenmillergroup.github.io/IMCDataAnalysis/index.html#citation.

6. https://bodenmillergroup.github.io/ImcSegmentationPipeline/segmentation.html.

7. Bankhead, P. et al. QuPath: Open source software for digital pathology image analysis. Scientific Reports (2017). https://doi.org/10.1038/s41598-017-17204-5.

8. https://cran.r-project.org/web/packages/Seurat/citation.html.

9. Schapiro, D., Jackson, H., Raghuraman, S. et al. histoCAT: analysis of cell phenotypes and interactions in multiplex image cytometry data. Nat Methods 14, 873–876 (2017). https://doi.org/10.1038/nmeth.4391.

10. SCIMAP development was led by Ajit Johnson Nirmal, Harvard Medical School. https://scimap.xyz.

Supplemental Figure Legends

Supplemental Figure 1. (A) Phenogram plot from a representative FL patient showing clusters identified using surface markers from Table S2. These clusters represented subsets of B cells, T cells, NK cells, monocytes and macrophages. (B) Graph showing percentage of individual surface marker-expressing cells from CD45^+^ cells in FL. (C) Graphs showing percentage of CD19^+^, CD3^+^, CD14^+^, CD56^+^ and CD16^+^ cells from CD45^+^ in different patient groups. (D) Heatmap showing surface expression using hierarchical clustering analysis. The 4 clusters representing 4 patient groups (G1-G4) were arbitrarily drawn based on phenotype related to T cells (CD3), B cells (CD19), monocytes/macrophaes (CD14) and NK cells (CD56). (E) Graphs showing percentage of T-cell markers from CD4^+^ (D) or CD8^+^ (E) T cells in different patient groups.

Supplemental Figure 2. (A) The tSNE plots of T cells from a representative FL patient showing identification of T_reg_, T_FH_, T_EXH_ (exhausted), T naïve and memory as well as SLEC and MPEC from CD4^+^ or CD8^+^ T cells. 18 subsets were identified by manual gating based on surface marker expression. (B, C) Kaplan-Meier curves for time to next treatment (TTNT) of FL patients (n = 82) by the number of S4, 5, 7, 10, 8, 9, 13 and 17 (B) as well as overall survival (OS) of CD45RA^+^ and CCR7^+^ T cells (C) with a cutoff of median number, respectively.

Supplemental Figure 3. (A-i) CITRUS plot showing clustering results from FL patients divided by 2 groups (early vs advanced stage). Circles in red represent clusters that differed between groups. Number in circles indicates a cluster ID. (A-ii), Graph showing quantitative results of abundance from all clusters. (A-iii), Histogram plots showing expression of selected markers by cells from all clusters overlaid on background staining. Expression level of each selected marker was expressed by cluster (red) over background (light blue). (B) Graphs showing percentage of surface marker-expressing cells from CD3^+^ T cells in tSNE-identified subsets presented in Figure 2D and Figure S2B.

Supplemental Figure 4. (A) Graphs showing expression level (mean fluorescent intensity, MFI) of CCR6 in patient groups identified in Figure 1C. (B) Graphs showing percentage of memory B cells (CD19^+^CD27^+^) from CD45^+^ cells in patient groups. (C) Dot plot from CITEseq analysis showing expression of surface markers CD19 and CCR6 to identify CD19^+^CCR6^+^ or CD19^+^CCR6^-^ B cells in FL. (D) Heatmap showing differential gene expression profile of CD19^+^CCR6^+^ when compared to CD19^+^CCR6^-^ B cells.

Supplemental Figure 5. (A) Kaplan-Meier curves for overall survival (OS) and time to next treatment (TTNT) of FL patients with by the number of CD3^+^ T cells with a cutoff of median number. (B) Graph showing percentage of T cells in CD45^+^ cells to stratify patients into groups (T-cell rich or poor). Patients with T cell numbers in the top or bottom 25% were grouped as rich or poor in T-cells. (C) The multi-dimensional scaling (MDS) plot showing dimension 1 vs 2 for CD45^+^ cells from patients with T-cell poor and rich. Each dot represents a patient. Calculations are based on the median (arcsinh-transformed) expression of 35 surface markers. Distances between samples in the plot approximate the typical change in medians. (D) CITRUS plot showing clustering (i), quantitative results of abundance (ii) and expression of selected markers (iii) from FL patients divided by 2 groups (poor or rich in T cells). (E) Graphs showing percentage of PD-1^high^, CD57^+^, 4-1BB^+^ and CD26^+^ cells in patients with either poor or rich in T cell number using median number as a cutoff point.

Supplemental Figure 6. (A) CITRUS plot showing clustering (i), quantitative results of abundance (ii) and expression of selected markers (iii) from FL patients divided by 2 groups (disease progression vs complete remission). (B) CITRUS plot showing expression of selected markers on clusters. (C) CITRUS plot showing clustering (i), quantitative results of abundance (ii) and expression of selected markers (iii) from FL patients divided by 2 groups (EFS24 failed vs achieved). (D) Dot plots showing CD57 expression on CD56^+^, CD19^+^, CD3^+^, CD14^+^, CD4^+^ and CD8^+^ cells from representative patient samples. FL, follicular lymphoma, PB: peripheral blood, LN: lymph node. (E) The tSNE plot showing CD57 expression on CD4^+^ (bottom) or CD8^+^ (top) T cells from a concatenated file (n=82). The dots in red and blue represent cells with or without CD57 expression, respectively. Graphs showing the percentage of CD57^+^ cells in CD4^+^ and CD8^+^ T cells (n=82). (F) The multi-dimensional scaling (MDS) plot showing dimension 1 vs 2 for the CD57^+^ T_FH_ and CD57^-^ T_FH_ from FL patients. Each dot represents a patient. Calculations are based on the median (arcsinh-transformed) expression of 23 surface markers. Distances between samples in the plot approximate the typical change in medians. (G) Graph showing fold change (log2) of surface marker expression in CD4^+^ or CD8^+^CD57^+^ versus CD4^+^ or CD8^+^CD57^-^ T_FH_ cells, respectively.

Supplemental Figure 7. (A) Data plots showing CD57 protein (CD57) and gene (B3GAT1) expression in FL using CITE-seq analysis. (B) Dot plots showing gating strategy to identify CD57^+^ or CD57^-^ T_FH_ cells using surface marker expression from CITEseq analysis in a representative FL patient (n=4). (C) Heatmap from a representative FL (n=4) showing differential gene expression of CD57^+^ T_FH_ cells compared to CD57^-^ T_FH_ cells using CITEseq analysis. (D) Histograms showing protein expression of GZMK and CD7 using flow cytometry as a validation of up- or down-regulated gene expression of GZMK and CD7, respectively, in CD57^+^ T_FH_ when compared to CD57^-^ T_FH_. (E) The violin plots showing gene and protein expression of CD161, CD27 and CXCR3 in CD57^+^ or CD57^-^ T_FH_ cells in FL. The gene and protein of each marker were measured by CITE-seq and CyTOF.


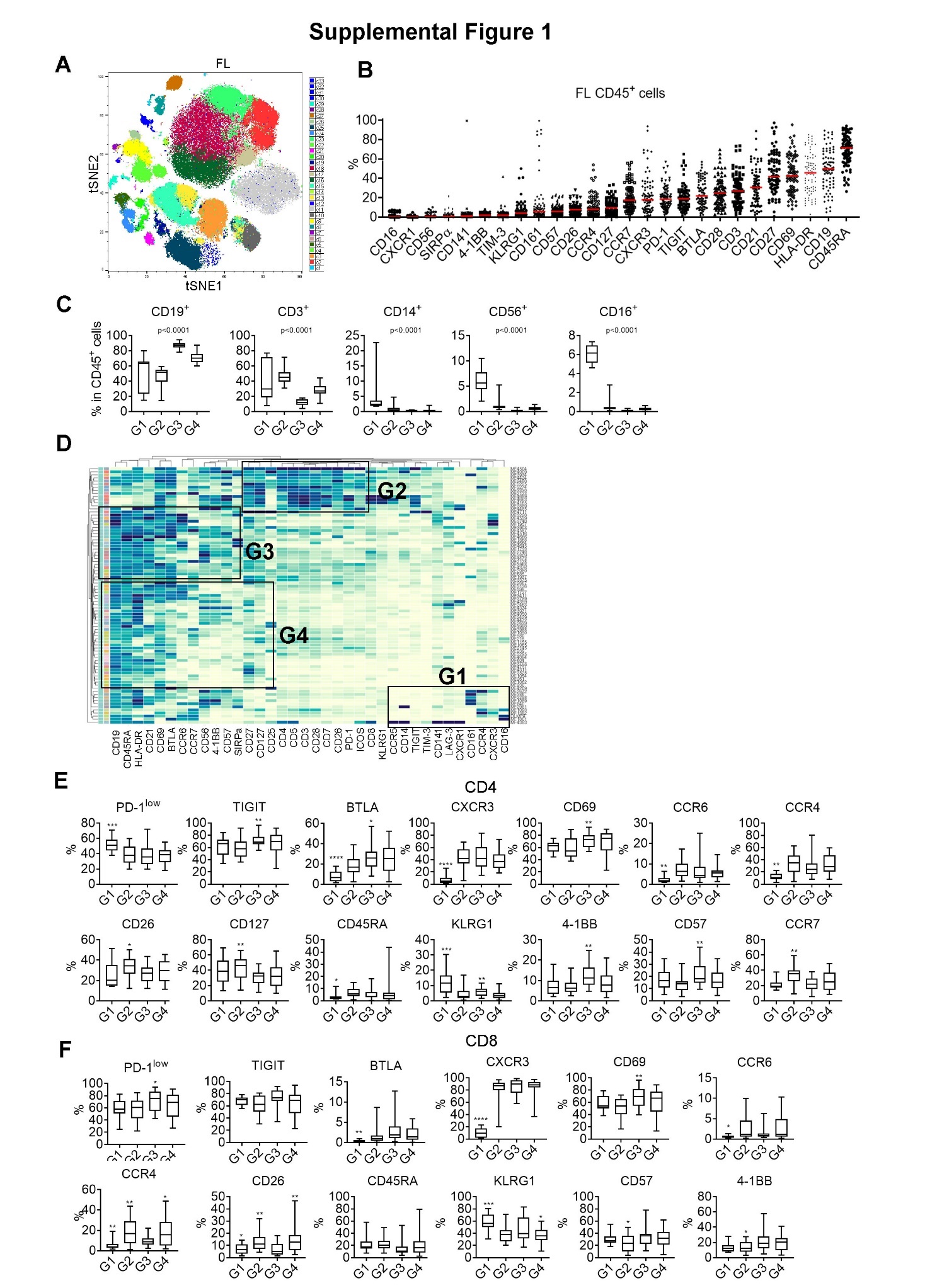


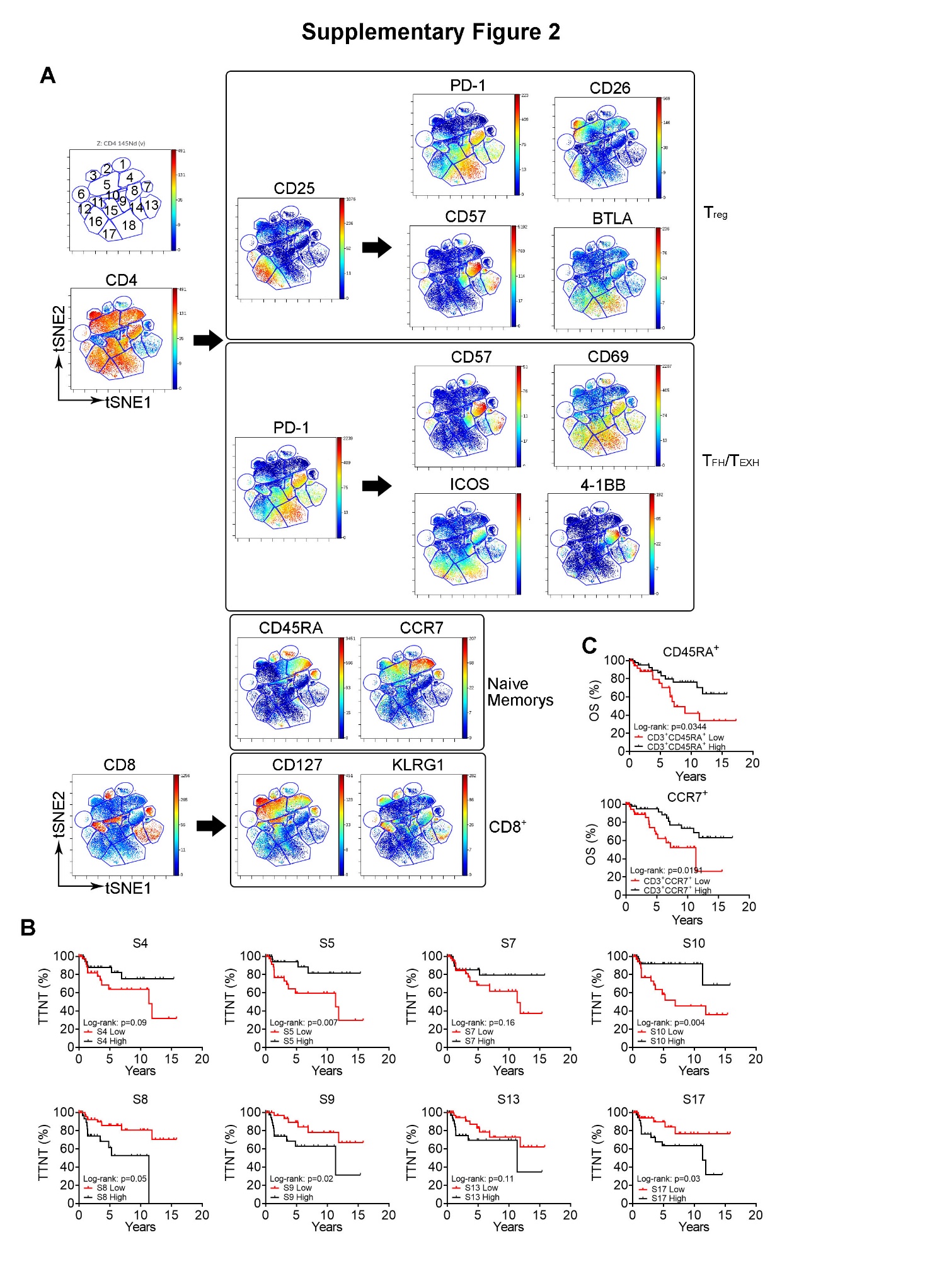


**
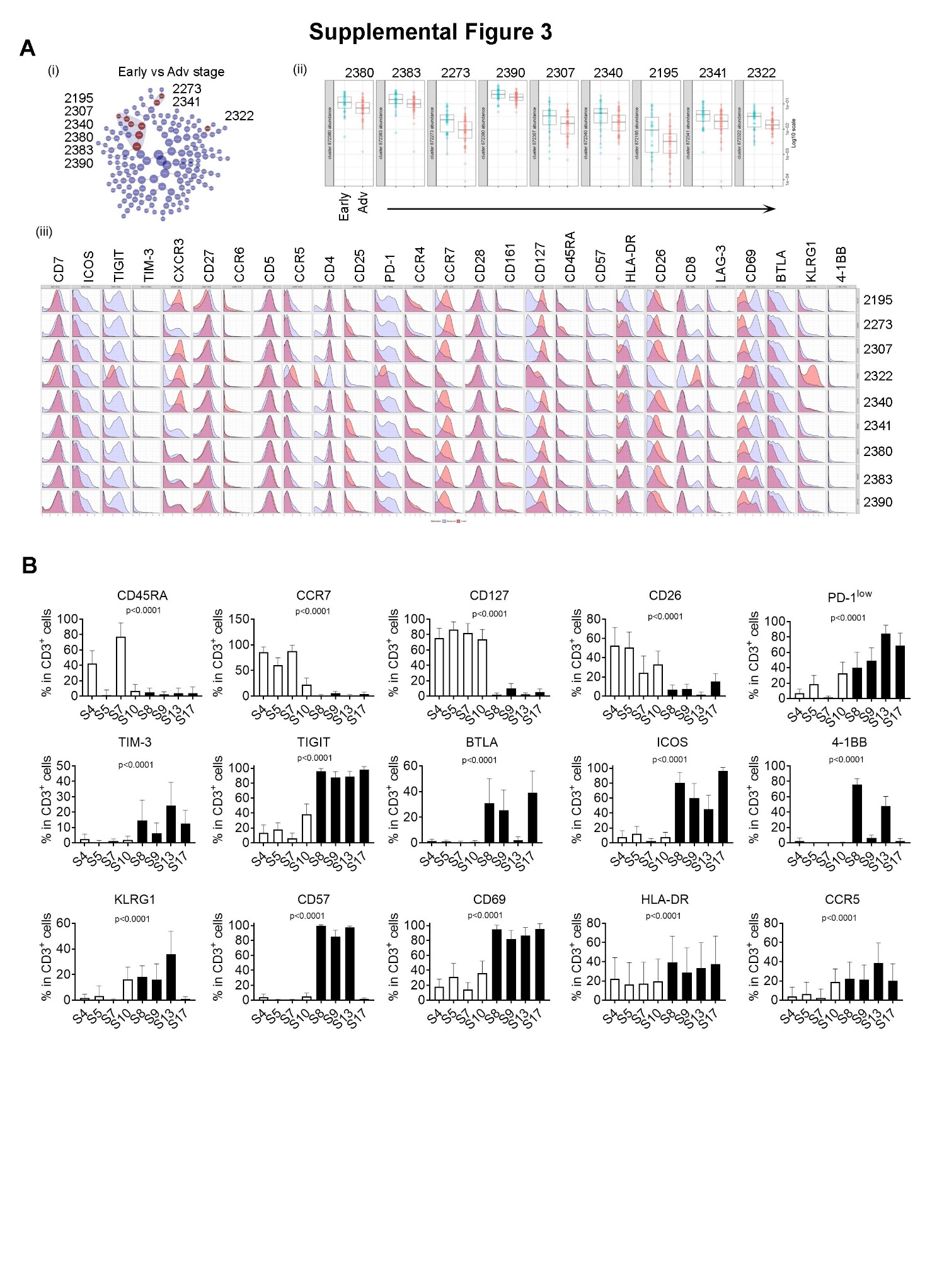
**

**
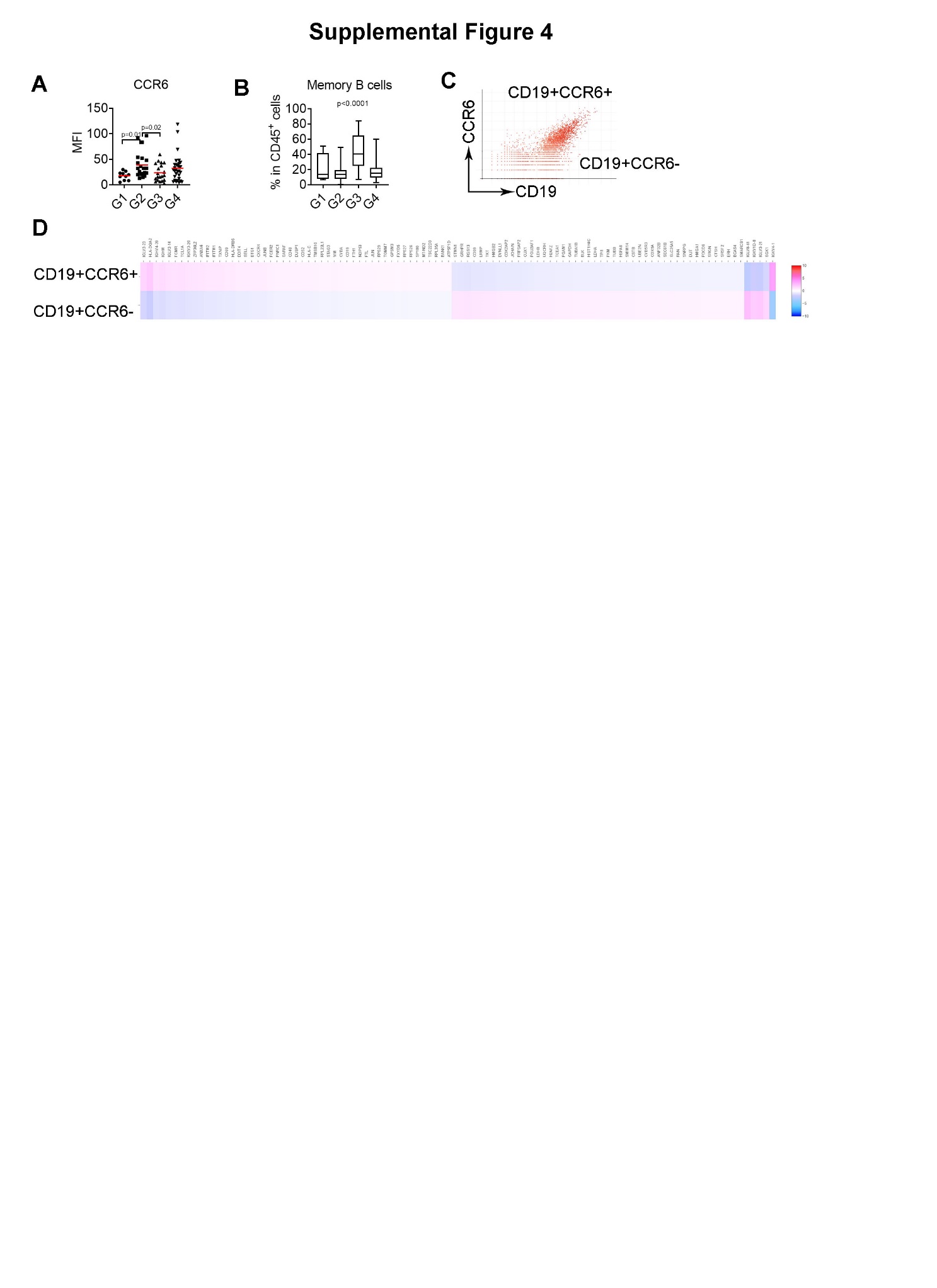
**

**
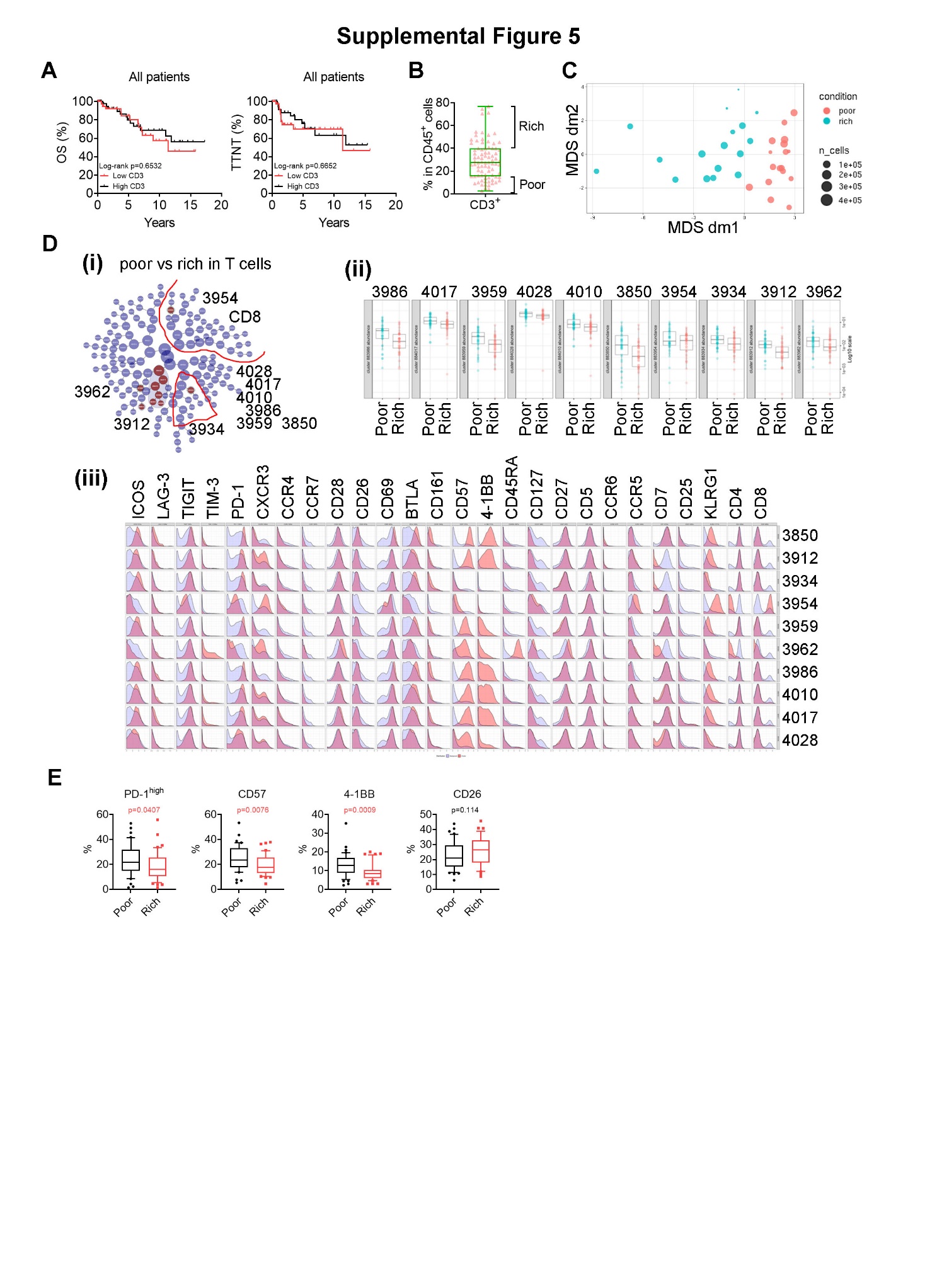
**

**
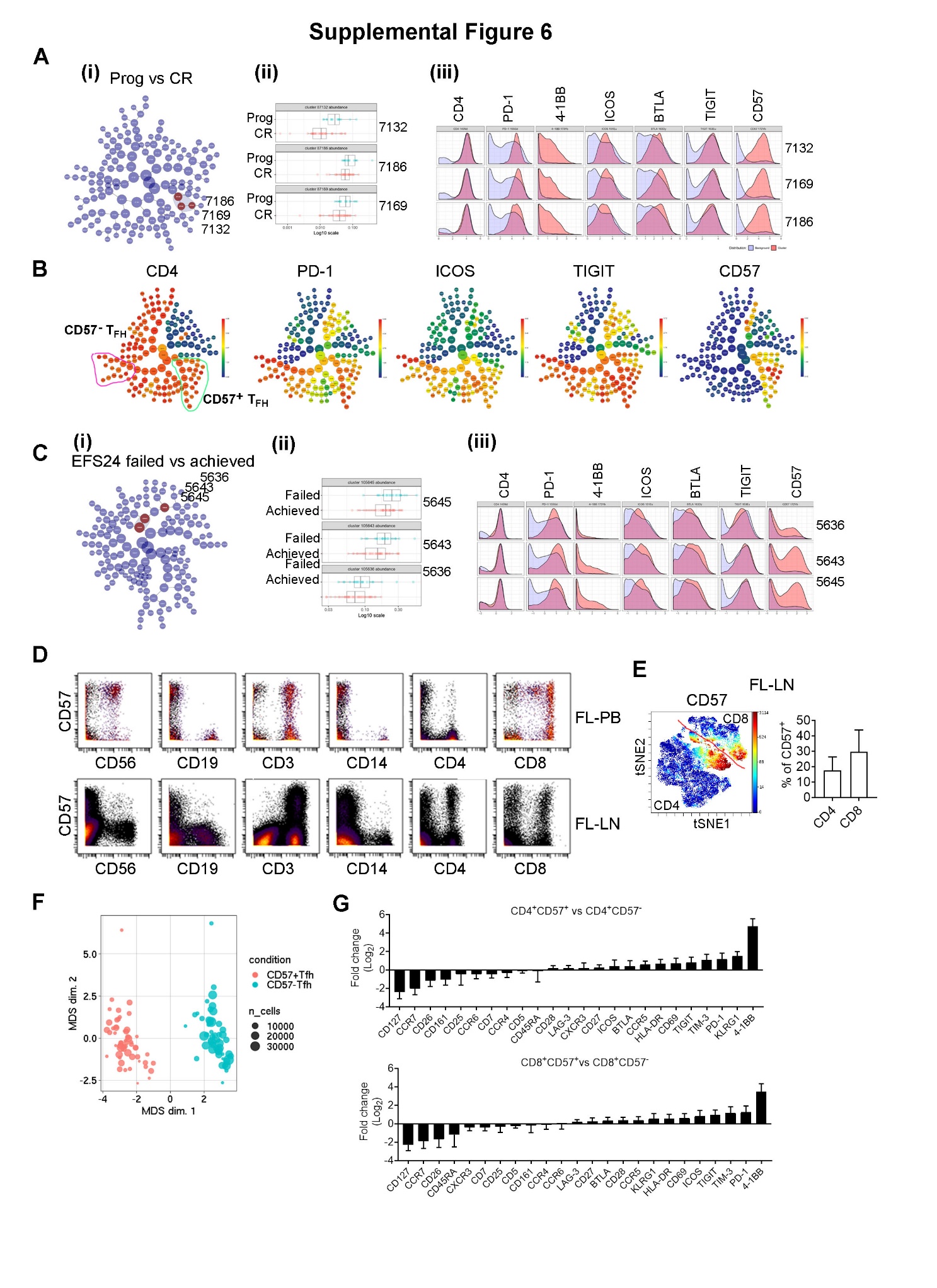
**

**
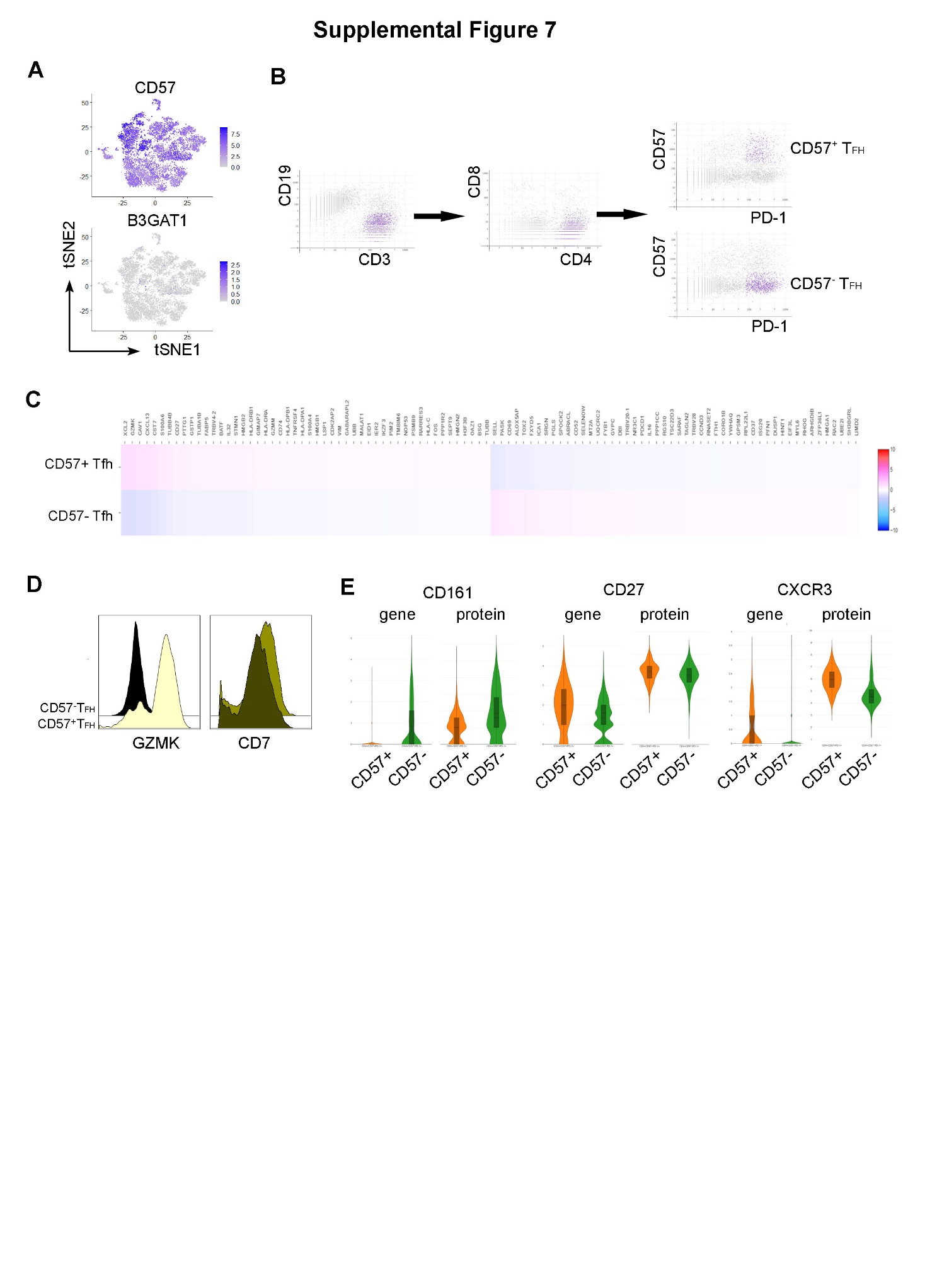
**

Supplementary Table 1. Patient characteristics (FL)

| Clinical parameters | n |
| --- | --- |
| Median age (64, range: 23-87, n=82) |  |
| Age<60 at diagnosis | 31 |
| Age>60 at diagnosis | 51 |
| Histology FL3a/b (n=81) |  |
| Yes | 20 |
| No | 61 |
| Stage III/IV (n=82) |  |
| Yes | 51 |
| No | 31 |
| B symptoms (n=81) |  |
| Yes | 6 |
| No | 75 |
| HGB<12 (n=81) |  |
| Yes | 17 |
| No | 64 |
| LDH abnormal (n=74) |  |
| Yes | 12 |
| No | 62 |
| FLIPI >3-5 (n=81) |  |
| Yes | 23 |
| No | 58 |
| Nodal>4 (n=80) |  |
| Yes | 24 |
| No | 56 |
| First line treatment (n=79) |  |
| Observation/Surgical remove | 35 |
| Rituximab/RT alone | 12 |
| Chemoimmunotherapy (CVP/R-CVP, R-CHOP, BR, LR-CD, Zevalin) | 32 |
| First line treatment response (n=76) |  |
| NR | 17 |
| SD | 10 |
| PR | 13 |
| CR | 36 |
| EFS24 (n=80) |  |
| Achieved | 42 |
| Failed | 23 |
| Not reached/Unknown | 15 |

Note: RT: Radiation therapy, NR: No response, SD: Stable disease, PR: Partial response, CR: Complete response; FL1, 2 or 3a: FL grade 1, 2 or 3a; R-CVP: cyclophosphamide, vincristine, prednisone) +rituximab; R-CHOP: cyclophosphamide, doxorubicin, vincristine, prednisone + rituximab. BR: Bendamustine + rituximab; LR-CD: lenalidomide, rituximab, cyclophosphamide, and dexamethasone

Supplementary Table 2. Staining Panel for CyTOF

| \|  \| **Target** \| **Clone** \| **Metal Tag** \| **Catalog #** \| \| --- \| --- \| --- \| --- \| --- \| \| 1 \| CD45 \| HI30 \| 89Y \| 3089003B \| \| 2 \| CD196 (CCR6) \| G034E3 \| 141Pr \| 3141003A \| \| 3 \| CXCR1 \| 8F1/CXCR1 \| 142Nd \| 3142009B \| \| 4 \| CD5 \| UCHT2 \| 143Nd \| 3143007B \| \| 5 \| CD195 (CCR5) \| NP-6G4 \| 144Nd \| 3144007A \| \| 6 \| CD4 \| RPA-T4 \| 145Nd \| 3145001B \| \| 7 \| CD8a \| RPA-T8 \| 146Nd \| 3146001B \| \| 8 \| CD7 \| CD76B7 \| 147Sm \| 3147006B \| \| 9 \| CD14 \| RMO52 \| 148Nd \| 3148010B \| \| 10 \| CD25 (IL-2R) \| 2A3 \| 149Sm \| 3149010B \| \| 11 \| CD223 (LAG3) \| 874501 \| 150Nd \| 3150016B \| \| 12 \| CD278, ICOS \| DX29 \| 151Eu \| 3151008B \| \| 13 \| CD21 \| BL13 \| 152Sm \| 3152010B \| \| 14 \| TIGIT \| MBSA43 \| 153Eu \| 3153001B \| \| 15 \| TIM-3 \| F38-2E2 \| 154Sm \| 3154010B \| \| 16 \| CD279 (PD-1) \| EH12.2H7 \| 155Gd \| 3155009B \| \| 17 \| CD183 (CXCR3) \| G025H7 \| 156Gd \| 3156004B \| \| 18 \| CD194 (CCR4) \| 205410 \| 158Gd \| 3158006A \| \| 19 \| CD197 (CCR7) \| G043H7 \| 159Tb \| 3159003A \| \| 20 \| CD28 \| CD28.2 \| 160Gd \| 3160003B \| \| 21 \| CD26 \| BA5B \| 161DY \| 3161015B \| \| 22 \| CD69 \| FN50 \| 162Dy \| 3162001B \| \| 23 \| CD272/BTLA \| MIH26 \| 163Dy \| 3163009B \| \| 24 \| CD161 \| HP-3G10 \| 164Dy \| 3164009B \| \| 25 \| CD19 \| HIB19 \| 165Ho \| 3165025B \| \| 26 \| CD141 \| M80 \| 166Er \| 3166017B \| \| 27 \| CD27 \| O323 \| 167Er \| 3167002B \| \| 28 \| CD127 (IL-7R) \| A019D5 \| 168Er \| 3168017B \| \| 29 \| CD45RA \| HI100 \| 169Tm \| 3169008B \| \| 30 \| CD3 \| UCHT1 \| 170Er \| 3170001B \| \| 31 \| KLRG1 \| 2F1 \| 171Yb \| Self-labeled \| \| 32 \| CD57 \| HCD57 \| 172Yb \| 3172009B \| \| 33 \| 4-1BB/CD137 \| 4B4-1 \| 173Yb \| 3173015B \| \| 34 \| HLA-DR \| L243 \| 174Yb \| 3174001B \| \| 35 \| SIRPa/CD172a \| SE5A5 \| 175Lu \| 3175024B \| \| 36 \| CD56 (NCAM) \| NCAM16.2 \| 176Yb \| 3176008B \| \| 37 \| CD16 \| 3GB \| 209BI \| 3209002B \| |
| --- | --- | --- | --- | --- | --- | --- | --- | --- | --- | --- | --- | --- | --- | --- | --- | --- | --- | --- | --- | --- | --- | --- | --- | --- | --- | --- | --- | --- | --- | --- | --- | --- | --- | --- | --- | --- | --- | --- | --- | --- | --- | --- | --- | --- | --- | --- | --- | --- | --- | --- | --- | --- | --- | --- | --- | --- | --- | --- | --- | --- | --- | --- | --- | --- | --- | --- | --- | --- | --- | --- | --- | --- | --- | --- | --- | --- | --- | --- | --- | --- | --- | --- | --- | --- | --- | --- | --- | --- | --- | --- | --- | --- | --- | --- | --- | --- | --- | --- | --- | --- | --- | --- | --- | --- | --- | --- | --- | --- | --- | --- | --- | --- | --- | --- | --- | --- | --- | --- | --- | --- | --- | --- | --- | --- | --- | --- | --- | --- | --- | --- | --- | --- | --- | --- | --- | --- | --- | --- | --- | --- | --- | --- | --- | --- | --- | --- | --- | --- | --- | --- | --- | --- | --- | --- | --- | --- | --- | --- | --- | --- | --- | --- | --- | --- | --- | --- | --- | --- | --- | --- | --- | --- | --- | --- | --- | --- | --- | --- | --- | --- | --- | --- | --- | --- | --- | --- | --- | --- | --- | --- |

|  | Patients with G1/2 | | | Patients with G3a/b | | |
| --- | --- | --- | --- | --- | --- | --- |
|  | Few T cells | More T cells | P value | Few T cells | More T cells | P value |
| Age>60 |  |  |  |  |  |  |
| Yes | 23 | 16 | 0.04 | 7 | 6 | 0.63 |
| No | 8 | 16 |  | 3 | 4 |  |
|  |  |  |  |  |  |  |
| Stage III/IV |  |  |  |  |  |  |
| Yes | 22 | 15 | 0.06 | 6 | 7 | 0.41 |
| No | 9 | 16 |  | 4 | 2 |  |
|  |  |  |  |  |  |  |
| B symptoms |  |  |  |  |  |  |
| Yes | 4 | 0 | 0.03 | 2 | 0 | 0.15 |
| No | 26 | 32 |  | 8 | 9 |  |
|  |  |  |  |  |  |  |
| EFS24 |  |  |  |  |  |  |
| Failed | 12 | 6 | 0.02 | 2 | 3 | 0.81 |
| Achieved | 10 | 20 |  | 6 | 7 |  |

Supplementary Table 3. Correlation between T cells with clinical parameters in FL patients with histology grade 1/2 or 3a/b

Cutoff point for few or more T cells is the median number of T cells among the patients in the cohort

Supplementary Table 4. Top 50 differentiated expressed genes (based on p value) of CD57^+^ T_FH_ cells vs CD57^-^ T_FH_ cells in FL

|  | Gene name | ENS ID | Log2 FC | P val |  | Gene name | ENS ID | Log2 FC | P val |
| --- | --- | --- | --- | --- | --- | --- | --- | --- | --- |
| 1 | GZMK | ENSG00000113088 | 2.008874 | 1.44E-187 | 26 | SRGN | ENSG00000122862 | -0.80219 | 2.10E-41 |
| 2 | CST7 | ENSG00000077984 | 1.709457 | 1.46E-143 | 27 | NR3C1 | ENSG00000113580 | -0.65494 | 1.80E-40 |
| 3 | CCL4 | ENSG00000275302 | 3.037206 | 5.08E-96 | 28 | CCL4L2 | ENSG00000276070 | 1.93457 | 4.22E-39 |
| 4 | CCL5 | ENSG00000271503 | 2.326377 | 1.86E-94 | 29 | UCP2 | ENSG00000175567 | -0.70072 | 2.12E-38 |
| 5 | IL32 | ENSG00000008517 | 1.282045 | 2.28E-94 | 30 | ATG7 | ENSG00000197548 | 0.546529 | 2.74E-37 |
| 6 | HMOX1 | ENSG00000100292 | 1.209019 | 5.61E-91 | 31 | SYNE2 | ENSG00000054654 | 0.810264 | 6.50E-37 |
| 7 | CD40LG | ENSG00000102245 | -1.4549 | 7.24E-91 | 32 | FCMR | ENSG00000162894 | 0.648431 | 1.80E-36 |
| 8 | GZMA | ENSG00000145649 | 1.996878 | 1.93E-87 | 33 | CHST12 | ENSG00000136213 | 0.691383 | 1.96E-35 |
| 9 | LAG3 | ENSG00000089692 | 1.890228 | 2.88E-82 | 34 | EOMES | ENSG00000163508 | 0.694502 | 3.26E-35 |
| 10 | CYTOR | ENSG00000222041 | 1.189506 | 9.45E-79 | 35 | FTH1 | ENSG00000167996 | -0.35672 | 7.93E-35 |
| 11 | F2R | ENSG00000181104 | 1.018613 | 4.95E-77 | 36 | RNF213 | ENSG00000173821 | 0.938277 | 1.43E-33 |
| 12 | NKG7 | ENSG00000105374 | 1.729106 | 6.58E-69 | 37 | IL10 | ENSG00000136634 | 0.647588 | 3.06E-33 |
| 13 | CADM1 | ENSG00000182985 | 0.952776 | 7.42E-69 | 38 | PTMS | ENSG00000159335 | 0.553616 | 4.26E-33 |
| 14 | PRF1 | ENSG00000180644 | 1.026662 | 1.16E-60 | 39 | RGS10 | ENSG00000148908 | -0.60691 | 6.77E-33 |
| 15 | CD81 | ENSG00000110651 | 0.847125 | 5.57E-59 | 40 | FKBP5 | ENSG00000096060 | -0.52205 | 1.37E-32 |
| 16 | SLAMF7 | ENSG00000026751 | 0.741867 | 1.25E-58 | 41 | TCF7 | ENSG00000081059 | -0.56019 | 2.19E-31 |
| 17 | CXCR6 | ENSG00000172215 | 0.937993 | 1.64E-57 | 42 | SMCO4 | ENSG00000166002 | -0.70168 | 2.52E-31 |
| 18 | CXCR3 | ENSG00000186810 | 0.979651 | 3.60E-55 | 43 | TUBB4A | ENSG00000104833 | 0.361377 | 1.74E-30 |
| 19 | CD27 | ENSG00000139193 | 0.671583 | 1.90E-51 | 44 | CAV1 | ENSG00000105974 | 0.848148 | 7.28E-30 |
| 20 | DUSP2 | ENSG00000158050 | 1.285957 | 3.92E-51 | 45 | CD79A | ENSG00000105369 | -1.28954 | 1.58E-29 |
| 21 | PRDM1 | ENSG00000057657 | 0.776209 | 4.86E-48 | 46 | HINT1 | ENSG00000169567 | -0.36732 | 2.24E-29 |
| 22 | PLEK | ENSG00000115956 | 0.776928 | 3.44E-46 | 47 | LHFPL6 | ENSG00000183722 | -0.61908 | 2.61E-29 |
| 23 | FYB1 | ENSG00000082074 | -0.68843 | 4.46E-45 | 48 | LIMS2 | ENSG00000072163 | -0.60211 | 2.73E-29 |
| 24 | MALAT1 | ENSG00000251562 | 0.555768 | 1.27E-43 | 49 | MIR4435-2HG | ENSG00000172965 | 0.657091 | 2.89E-29 |
| 25 | FXYD5 | ENSG00000089327 | -0.66788 | 2.88E-43 | 50 | EEF1A1 | ENSG00000156508 | -0.22034 | 4.09E-29 |

**Supplementary Table 5. Staining panel for Imaging Mass Cytometry (Hyperion)**

| **Target Name** | **Hyperion Metal Tag** | **Hyperion Clone** | **Cat#** | **Dilution** |
| --- | --- | --- | --- | --- |
| Alpha-SMA | 141Pr | 1A4 | 3141017D | 1:200 |
| CD19 | 142Nd | 6OMP31 | 3142014D | 1:400 |
| Vimentin | 143Nd | D21H3 | 3143027D | 1:100 |
| CD14 | 144Nd | EPR3653 | 3144025D | 1:200 |
| CD16 | 146Nd | EPR16784 | 3146020D | 1:100 |
| Pan-Keratin | 148Nd | C11 | 3148020D | 1:200 |
| CD11b | 149Sm | EPR1344 | 3149028D | 1:100 |
| CD45 | 152Sm | D9M8I | 3152018D | 1:100 |
| CD11c | 154Sm | EP1347Y | Mayo-ab216655 | 1:400 |
| FoxP3 | 155Gd | PCH101 | 3155018D | 1:100 |
| CD4 | 156Gd | EPR6855 | 3156033D | 1:400 |
| E-Cadherin | 158Gd | 24E10 | 3158029D | 1:50 |
| CD68 | 159Tb | KP1 | 3159035D | 1:100 |
| Vista | 160Gb | D1L2G | 3160025D | 1:50 |
| CD20 | 161Dy | H1 | 3161029D | 1:800 |
| CD8a | 162Dy | C8/144B | 3162034D | 1:100 |
| CD45RA | 166Er | HI100 | Mayo-304102 | 1:100 |
| Granzyme B | 167Er | EPR20129-217 | 3167021D | 1:50 |
| Ki67 | 168Er | B56 | 3168022D | 1:50 |
| Collagen type 1 | 169Tm | Polyclonal | 3169023D | 1:600 |
| CD3 | 170Er | Polyclonal | 3170019D | 1:100 |
| Histone H3 | 171Yb | D1H2 | 3171022D | 1:600 |
| CD45RO | 173Yb | UCHL1 | 3173016D | 1:50 |
| HLA-DR | 174Yb | LN3 | 3174025D | 1:100 |
| Beta-2 Microglobulin | 175Lu | B2M961 | Mayo-MAB1430 | 1:200 |
| Na/K ATPase | 176Yb | EP1845Y | Mayo-NBP23452801 | 1:200 |
